# Genome-wide identification of *U-box* gene family and expression analysis in response to saline-alkali stress in foxtail millet (*Setaria italica* L. Beauv)

**DOI:** 10.3389/fgene.2024.1356807

**Published:** 2024-02-16

**Authors:** Xiaoke Zhou, Yun Li, Jian Wang, Yuxue Zhao, Huimin Wang, Yucui Han, Xiaohu Lin

**Affiliations:** ^1^ Hebei Key Laboratory of Crop Stress Biology, College of Agronomy and Biotechnology, Hebei Normal University of Science and Technology, Qinhuangdao, China; ^2^ Research Center of Rural Vitalization, Hebei Normal University of Science and Technology, Qinhuangdao, China

**Keywords:** foxtail millet, *U-box*, gene family, saline-alkali stress, qRT-PCR

## Abstract

E3 ubiquitin ligases are central modifiers of plant signaling pathways that regulate protein function, localization, degradation, and other biological processes by linking ubiquitin to target proteins. E3 ubiquitin ligases include proteins with the U-box domain. However, there has been no report about the foxtail millet (*Setaria italica* L. Beauv) *U-box* gene family (*SiPUB*) to date. To explore the function of *SiPUBs*, this study performed genome-wide identification of *SiPUBs* and expression analysis of them in response to saline-alkali stress. A total of 70 *SiPUBs* were identified, which were unevenly distributed on eight chromosomes. Phylogenetic and conserved motif analysis demonstrated that *SiPUBs* could be clustered into six subfamilies (I–VI), and most *SiPUBs* were closely related to the homologues in rice. Twenty-eight types of *cis*-acting elements were identified in *SiPUBs*, most of which contained many light-responsive elements and plant hormone-responsive elements. Foxtail millet had 19, 78, 85, 18, and 89 collinear *U-box* gene pairs with *Arabidopsis*, rice, sorghum, tomato, and maize, respectively. Tissue specific expression analysis revealed great variations in *SiPUB* expression among different tissues, and most *SiPUBs* were relatively highly expressed in roots, indicating that *SiPUBs* may play important roles in root development or other growth and development processes of foxtail millet. Furthermore, the responses of 15 *SiPUBs* to saline-alkali stress were detected by qRT-PCR. The results showed that saline-alkali stress led to significantly differential expression of these 15 *SiPUBs*, and *SiPUB20/48/70* may play important roles in the response mechanism against saline-alkali stress. Overall, this study provides important information for further exploration of the biological function of *U-box* genes.

## 1 Introduction

Ubiquitin/26S proteasome pathway is one of the most important protein degradation pathways in cells (Wang et al., 2018). This pathway can regulate all aspects of plant growth and development and the degradation of short-lived regulatory proteins (Santner and Estelle, 2010). Ubiquitin proteasome degradation pathway consists of ubiquitin (Ub), ubiquitin activing enzyme (E1), ubiquitin conjugating enzyme, E2), ubiquitin protein ligating enzyme (E3), 26S proteasome (proteasome), deubiquitinating enzymes (DUBs), and protein substrates (March and Farrona, 2018). Ubiquitin is a very important molecule in this pathway. As a marker protein, ubiquitin participates in the processes of recognition, labeling, and targeted degradation of proteins. Ubiquitin ligase E3 is required for ubiquitin activation and transfer and plays a key role in protein ubiquitination (Trujillo, 2018). Ubiquitin ligase E3 finally binds ubiquitin to the target protein to form ubiquitinated target protein. These ubiquitination targets are then snipped into small peptide chains or free amino acids by the proteasome. Among the enzymes in the ubiquitin degradation pathway, ubiquitin ligase E3 has the highest variety and quantity, including Really Interesting New Gene (RING), *U-box* domain protein, and homology to the E6AP C-Terminus (HECT) (Mandal et al., 2018).

*U-box* is a protein domain playing an important role in the ubiquitin/26S proteasome pathway. It was first discovered in yeast Ub Fusion Degradation 2 (UFD2) (Van et al., 2022). The *U-box* domain is a unique domain composed of about 70 amino acids and transfers ubiquitin from E2s to target proteins through salt bridges, ion chelation, and hydrogen bonding (Qi et al., 2017). It can recognize and bind target proteins and attach ubiquitin to these target proteins, thereby targeting them into ubiquitinated proteins that participate in the cell degradation process. Previous studies have found a large number of *U-box* genes in many species, and many studies have reported that *U-box* genes are responsive to abiotic stresses such as light, drought, and salt in a variety of plants, indicating that *U-box* gene family plays an important role in different stages of plant growth (Bergler and Hoth, 2011; Woodson et al., 2015; Adler et al., 2017; Wang et al., 2020b). Therefore, identifying the unique roles of different *U-box* proteins in different stress processes will help us understand the development of plant resistance (Li et al., 2017; Kim et al., 2021; Tang et al., 2022).

Foxtail millet (*Setaria italica* L. Beauv), which belongs to the Poaceae family, is one of the oldest cultivated crops in China. It was domesticated from wild foxtail grass and has a planting history of approximately 10,000 years, playing a crucial role in the agricultural civilization history of arid regions in northern China (Diao and Jia, 2017). As the main cultivated crop in dry green agriculture, it has the characteristics of small genome, short life cycle, self-pollination, drought resistance, and can grow under low fertility conditions, making it a model plant for C4 cereal crop research (Muthamilarasan and Prasad, 2014; Yang et al., 2020). With the completion of foxtail millet genome sequencing in 2012 and release of related sequence information, there has been increasing genome-based research on foxtail millet breeding (Bennetzen et al., 2012). Environmental stress has adverse effects on plant growth and development (Cramer et al., 2011). Excessive salinity in the soil has a great negative impact on plant growth and productivity, leading to large reduction in grain yield (Shrivastava and Kumar, 2015). Current studies have shown that *U-box* gene plays an important role in resistance of rice, sorghum and other plants (Hu et al., 2018; Yang et al., 2021; Fang et al., 2022). For example, plants overexpressing *OsPUB15* in rice have higher salt tolerance than the wild type (Park et al., 2011). In *Arabidopsis*, *PUB13* inactivation leads to elevated concentrations of the defense hormone salicylic acid, spontaneous cell death, and early flowering (Trujillo, 2018). In wheat, *TaPUB1* induces the expression of target genes, thereby enhancing antioxidant capacity under stress conditions (Wang et al., 2020b). Overexpression of the *GmPUB8* gene during seed germination and post-germination growth stages leads to hypersensitivity to salt and drought stress in soybean (Wang et al., 2016).The *U-box* gene family may play an important role in foxtail millet stress response. So far, there has been no report about the identification and expression analysis of *U-box* gene family (*SiPUB*) in foxtail millet. In this study, *SiPUBs* were identified and their expression was analyzed under saline-alkali stress, and the results may provide an important theoretical basis for the functional analysis of *U-box* genes.

## 2 Materials and methods

### 2.1 Plant materials and treatments

The salt-alkali tolerant foxtail millet variety, Jikegu3 (JK3), and the salt-alkali sensitive foxtail millet variety, Bao175 (B175), were selected as experimental materials based on previous studies. Seeds of uniform size were selected and sterilized with NaClO for 5 min, washed with distilled water 5 times, soaked in distilled water at room temperature for 24 h, sown, placed in artificial climate tank (Day/night duration: 12 h/12 h; Day/night temperature: 28°C/22°C; Humidity: 65%) and cultured until Sanye One stage, and 75% seawater (Bohai Sea water, water taken from the coast of Qinhuangdao Sea Port Area, China) were treated with salt and alkali stress. Both materials were sampled at 0, 6, 12, 24, and 48 h after treatment, and a mixture of leaves (second and third leaves) from the plants was collected. Each treatment was replicated three times. The samples were placed in sterile 5-mL centrifuge tubes, labeled, immediately frozen in liquid nitrogen, and stored at −80°C until RNA extraction.

### 2.2 Identification of SiPUB members

The genome and protein sequences of foxtail millet were obtained from the Ensemble database (https://asia.ensembl.org/index.html). The gene sequences and protein sequences of *Arabidopsis U-box* gene family were downloaded from the *Arabidopsis* database (http://www.arabidopsis.org/browse/genefamily/pub.jsp). Pfam (http://pfam.xfam.org/), BLAST, SMART (http://smart.embl.de/smart/batch.pl), and NCBI-CDD (https://www.ncbi.nlm.nih.gov/cdd) online databases were used to confirm the presence of complete conserved domains.

First, all *U-box* domain sequences (PF04564) from different species were downloaded from the Pfam database (http://pfam.xfam.org/). A hidden Markov model (HMM) was constructed using Hmmer2.3.2 (http://hmmer.janelia.org/) and used to search for *U-box* gene members in the foxtail millet protein database on Ensemble, with a condition of an E-value lower than 1 × 10^−5^. Then, 64 *Arabidopsis U-box* gene sequences and protein sequences were downloaded from the TAIR database (https://www.arabidopsis.org/), and a BLAST comparison was performed using TBtools software. The intersection of the BLAST alignment results and HMM results yielded the gene and protein sequences of the *U-box* gene family. Based on the above results, NCBI-CDD database (https://www.ncbi.nlm.nih.gov/Structure/bwrpsb/bwrpsb.cgi) and SMART website (http://smart.embl-heidelberg.de/) were used for domain prediction. The sequences that did not contain the *U-box* domain were removed, resulting in the identification of all *U-box* members. Physicochemical properties such as molecular weight and isoelectric point were analyzed by TBtools, and subcellular localization was analyzed by WOLF (https://wolfpsort.hgc.jp/).

The resulting *U-box* family members were named according to their Chromosomal position, such as *SiPUB1*: “Si” represents *S. italica*, “PUB” is the abbreviation for the gene family, and “1”denotes the sequence number based on their position on the Chromosomal.

### 2.3 Gene structure and chromosomal localization of SiPUBs

The DNA sequences of identified *SiPUBs* were downloaded from the ensemble database. GSDS online software (http://gsds.gao-lab.org/index.PHP) and TBtools analysis software (https://github.com/CJChen/TBtools) were used to respectively detect the structural and conserved motifs of *SiPUB* exons and introns. At the same time, gene location information was obtained, and the chromosomal mapping of genes was presented using MapChart software.

### 2.4 Systematic evolution and protein domain analysis of SiPUBs

MEGA_11.0.13 was applied to perform multiple sequence alignment of protein sequences in *SiPUBs*, and the phylogenetic tree was constructed by NJ (Neighbor-Joining) method. The protein domains of SiPUBs were analyzed using the online tool SMART.

### 2.5 Promoter analysis and gene ontology annotation of SiPUBs

The 2000bp sequence upstream of *SiPUB* promoter was extracted and uploaded to the *SiPUB* PlantCARE (http://bioinformatics.psb.ugent.be/webtools/plantcare/html/) web site for *cis*-element prediction. Gene ontology (GO) annotation for *SiPUBs* was performed using R 4.3.1.

### 2.6 Analysis of collinearity and duplication events

The *U-box* protein sequences of *Arabidopsis*, rice, sorghum, maize, tomato and foxtail millet were analyzed by interspecific and intraspecific collinearity, and the gene duplication events of foxtail millet were analyzed by MCScanX. TBtools was used to visualize the results.

### 2.7 Protein-protein interaction

The functional protein-protein interaction network model of *U-box* proteins was integrated using Web String (https://string-db.org/), with the default confidence parameter value of 0.400.

### 2.8 Tissue expression pattern of SiPUBs

The tissue-specific expression patterns of *SiPUBs* were studied by NCBI Short Read Archive database (https://www.ncbi.nlm.nih.gov/sra/) access to different groups of the transcriptome data. Heat map analysis was used in TBtools to map gene expression heat maps using log2 (TPM +1) scales.

### 2.9 Total RNA extraction and cDNA synthesis

Total RNA was extracted using the SteadyPure Plant RNA Extraction Kit from Accurate Biotechnology (Hunan) Co., Ltd. The quality and concentration of RNA were evaluated using a micro-volume nucleic acid and protein analyzer and RNase-free agarose gel electrophoresis. After confirming the quality and concentration of RNA, the Evo M-MLV Reverse Transcription Premix Kit from Accurate Biotechnology (Hunan) Co., Ltd. was employed to reverse transcribe it into cDNA following the instructions. The cDNA was then stored at −20°C for future use.

### 2.10 RT-qPCR analysis

Gene primers were designed using Primer Premier 6, with foxtail millet EF-1a gene serving as the internal reference gene. The primer specificity was screened through BLAST website comparison, and the alignment data were obtained from NCBI (Table 1). The qPCR system was prepared using the SYBR Green Premix Pro Taq HS qPCR Kit from Accurate Biotechnology (Hunan) Co., Ltd. The qPCR system was run in a fluorescence quantitative PCR instrument. To ensure the accuracy of the results, three biological replicates and three technical replicates were performed. The relative expression levels of RNA transcripts were calculated using the 2^−ΔΔCT^ method.

**TABLE 1:** Primers for RT-qPCR.

Gene	Gene-id (mg)	Forward primer (5′–3′)	Reverse primer (5′–3′)
*EF-1a*	SETIT_026288	TGA​CTG​TGC​TGT​CCT​CAT​CA	TGA​CTG​TGC​TGT​CCT​CAT​CA
*SiPUB1*	SETIT_016453	GTC​GTC​GGA​TTG​GAT​GGC​ACA​G	CCG​TAG​TCC​ACC​CAT​TCT​TCA​ACA​C
*SiPUB14*	SETIT_016624	ATC​CTG​TCA​GTT​CTT​GTG​AGC​CAT​C	AAT​AGC​AGC​AGC​ATT​CTC​CTT​GTT​G
*SiPUB18*	SETIT_028897	GTT​GCC​ACC​AGT​GTC​ACC​TCA​G	ACC​GCA​GCC​TCC​TCG​TCA​AT
*SiPUB19*	SETIT_029006	CAC​AGT​CGC​AGT​TCA​GTC​AAG​AGG	GCA​AGC​CAG​ACG​GTC​ATC​CAA​G
*SiPUB20*	SETIT_029090	GAA​TGC​TCC​GAG​GAG​ATT​GCC​TTC	GCA​GTA​CAC​CAG​GAA​CGC​TAT​CAA​G
*SiPUB21*	SETIT_032533	CAC​CAT​TCG​CAC​AAT​CGT​CAA​GTT​C	ACC​GCT​CCA​TTC​AAT​TCG​CTT​ATC​T
*SiPUB24*	SETIT_022917	CGC​TGC​TCG​ACA​AGG​AGG​AAT​T	TTC​GCA​AGG​ACC​TGC​CAA​ATG​T
*SiPUB26*	SETIT_022018	CGG​AGC​TGG​TCG​CTT​ATC​TCA​C	GAG​TAA​CGA​CGA​CGG​CAT​CCT​TAG
*SiPUB36*	SETIT_005898	ATC​GCA​CCA​AGC​CAC​TTC​ATC​TG	TGG​AAG​CCT​CTG​ATT​GTT​GAC​CG
*SiPUB43*	SETIT_013258	GGT​GGC​AGT​CCT​CTA​CAT​CCT​AGT	AGT​TCT​GAT​TGG​CGA​CAC​TCT​GG
*SiPUB48*	SETIT_015737	CCG​ATG​AGG​TCA​AGG​AGC​AAG​TG	GTT​CCT​CTC​GTC​CTC​GTT​GTT​CTG
*SiPUB53*	SETIT_009444	TCG​CAA​GTC​GGA​GAA​GGA​AGA​GAT	AGT​TGA​GGA​TGC​CGT​CGT​GGA​T
*SiPUB55*	SETIT_011652	AGT​GGC​TAG​ACA​GAG​GCT​TCA​GG	CGG​TGC​TTC​CAG​AGA​CGA​GAT​TC
*SiPUB58*	SETIT_034460	TGA​GGA​CCA​TGG​CAA​GTG​TC	CTC​AAG​TGC​ATC​AAT​CGG​CG
*SiPUB70*	SETIT_034816	ACG​GAG​CCA​GAT​GAG​GAA​GAA​GAA	TTG​TTG​AAC​CAG​CGA​CAC​CAG​TT

### 2.11 Protein structure analysis

Protein structure analysis was carried out for *SiPUBs*, and secondary structure analysis of proteins in *SiPUBS* was performed using SOPMA (https://npsa-prabi.ibcp.fr/cgi-bin/npsa_automat.pl?page=npsa_sopma.html). The tertiary structure was predicted based on homology modeling method of online software SWISS-MODEL (http://swissmodel.expasy.org/interactive).

## 3 Results

### 3.1 Identification and physicochemical property analysis of SiPUBs

By using the *Arabidopsis U-box* gene family protein sequences as references, we conducted a BLAST comparison with the foxtail millet genome to screen candidate *U-box* genes, and 2016 gene family members were obtained. The Hidden Markov Model (HMM) file (PF04564) of the *U-box* family was obtained from the Pfam database and the conserved protein sequences of the species were summarized, and 79 gene family members were obtained. By combining the results of the two methods, 78 gene family members were obtained. Protein sequence domains were detected using SMART and CDD, and redundancies and deletions were removed. Finally, 70 *U-box* genes with complete *U-box* domains were identified (Supplementary Table S1), which were named as *SiPUB1*–*SiPUB70* according to their position on chromosomes. The 70 *SiPUBs* showed great variations in protein sequences and physicochemical properties, with the amino acid (aa) length ranging from 275 aa (*SiPUB45*) to 1029 aa (*SiPUB65*) and the molecular weight ranging from 30946.21 Da to 115391.56 Da. The isoelectric point (pI) ranged from 5.17 to 8.98, but was lower than 7 for most proteins, indicating that most SiPUBs are rich in acidic amino acids and belong to acidic proteins. The instability index ranged from 32.99 to 68.16, and the aliphatic index was from 68.17 to 111.56. The most hydrophilic protein was *SiPUB9* (−0.617), and the most hydrophobic protein was *SiPUB59* (0.317). An online WOLF server was used to predict the localization of 70 SiPUBs in cells. The results showed different locations of SiPUBs in cells, and most of them were located in chloroplasts and cytoplasm. The subcellular localization results indicated that SiPUBs play a key role in biological processes such as plant growth and development.

### 3.2 Chromosomal localization of SiPUBs

**FIGURE 1:**
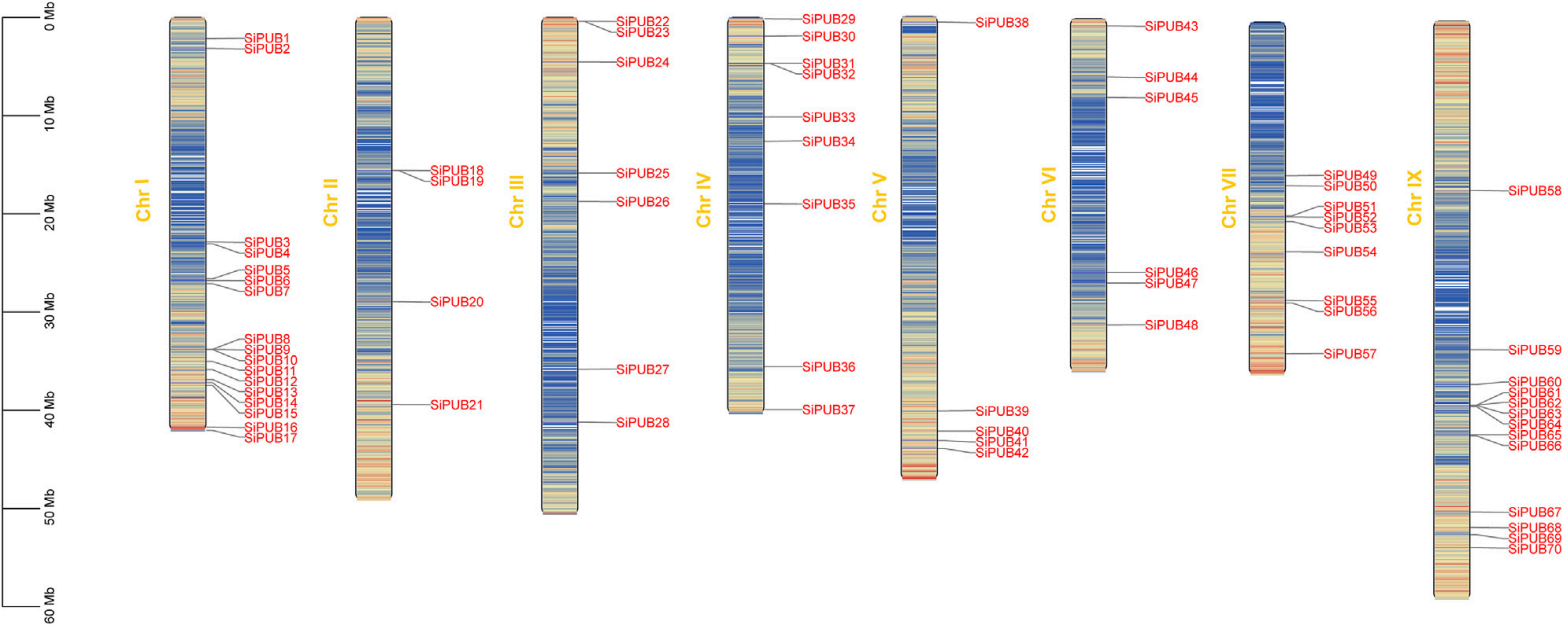
Chromosomal localization of *SiPUBs*. The varying colors of chromosomes indicate gene density.

Based on the information in a chromosomal distribution map of *SiPUBs* was obtained using the MapChart software. Figure 1 shows that *SiPUBs* are distributed on eight chromosomes in an uneven manner. Among different chromosomes, chromosome I had the largest number of *SiPUBs* (up to 17), followed by chromosome IX (13), while chromosome II had the fewest *SiPUBs* (only 4). Moreover, five tandem gene clusters involving 12 genes were found in foxtail millet genome, which were distributed on chromosomes I, II, III, IV, and IX, respectively, but no tandem gene cluster was found on chromosomes V, VI, and VII.

### 3.3 Secondary structure analysis of SiPUBs

As shown in Supplementary Table S2, the secondary structure of SiPUBs includes alpha-helix, ex-tended strand, beta-sheet, and random coil. Alpha-helix (26.97%–70.86%) and random coil (21.58%–44.52%) were relatively more abundant in the protein sequences, while extended strand (2.70%–13.92%) and beta-sheet (1.36%–7.73%) were relatively less abundant.

### 3.4 Phylogenetic analysis of SiPUBs

**FIGURE 2:**
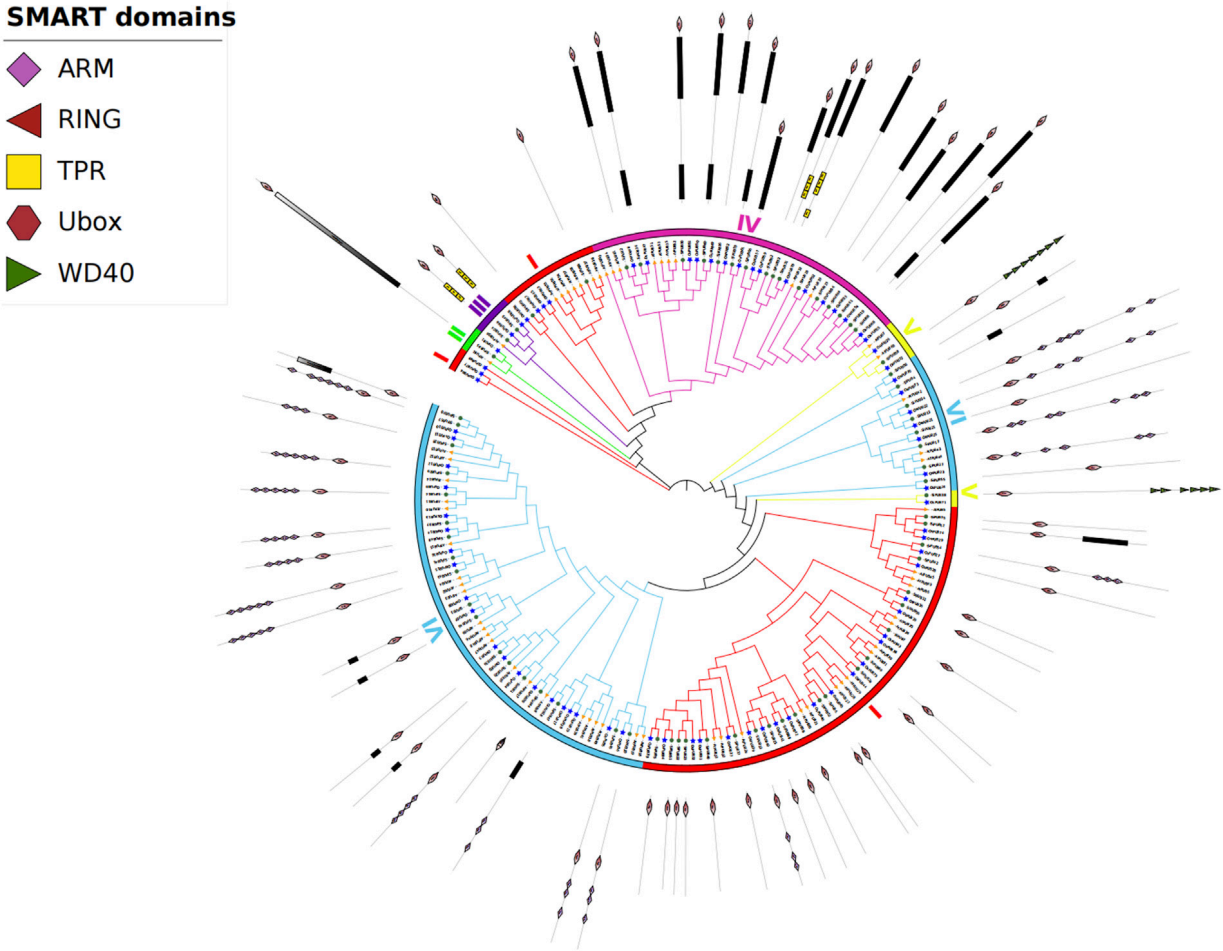
Phylogenetic tree of *U-box* proteins from foxtail millet, *Arabidopsis,* and rice. Different colors indicate different subfamilies. The three species are represented by three different shapes.

To study the evolutionary relationship of SiPUB proteins, a neighbor-joining (NJ) tree was constructed with *U-box* proteins from foxtail millet (70 proteins), rice (77 proteins) and *Arabidopsis* (61 proteins). According to the phylogenetic relationships and domain composition, these proteins could be divided into six subfamilies, including *U-box* only (I), UFD2 specific motif + *U-box* (II), TPR + *U-box* (III), Kinase + *U-box* (IV), *U-box* + WD40 (V), and *U-box* + ARM (VI). Subfamilies I, II, III, IV, V, and VI contained 23, 1, 2, 16, 2, and 26 members, respectively (Figure 2). Of the six subfamilies, subfamily VI (*U-box* + ARM) included the most genes (26 genes). In general, the *U-box* genes of foxtail millet were more closely related to homologues in rice than to those in *Arabidopsis*, and the *U-box* genes with similar genetic structures were clustered together.

### 3.5 Gene structure and conserved motif analysis of SiPUBs

**FIGURE 3:**
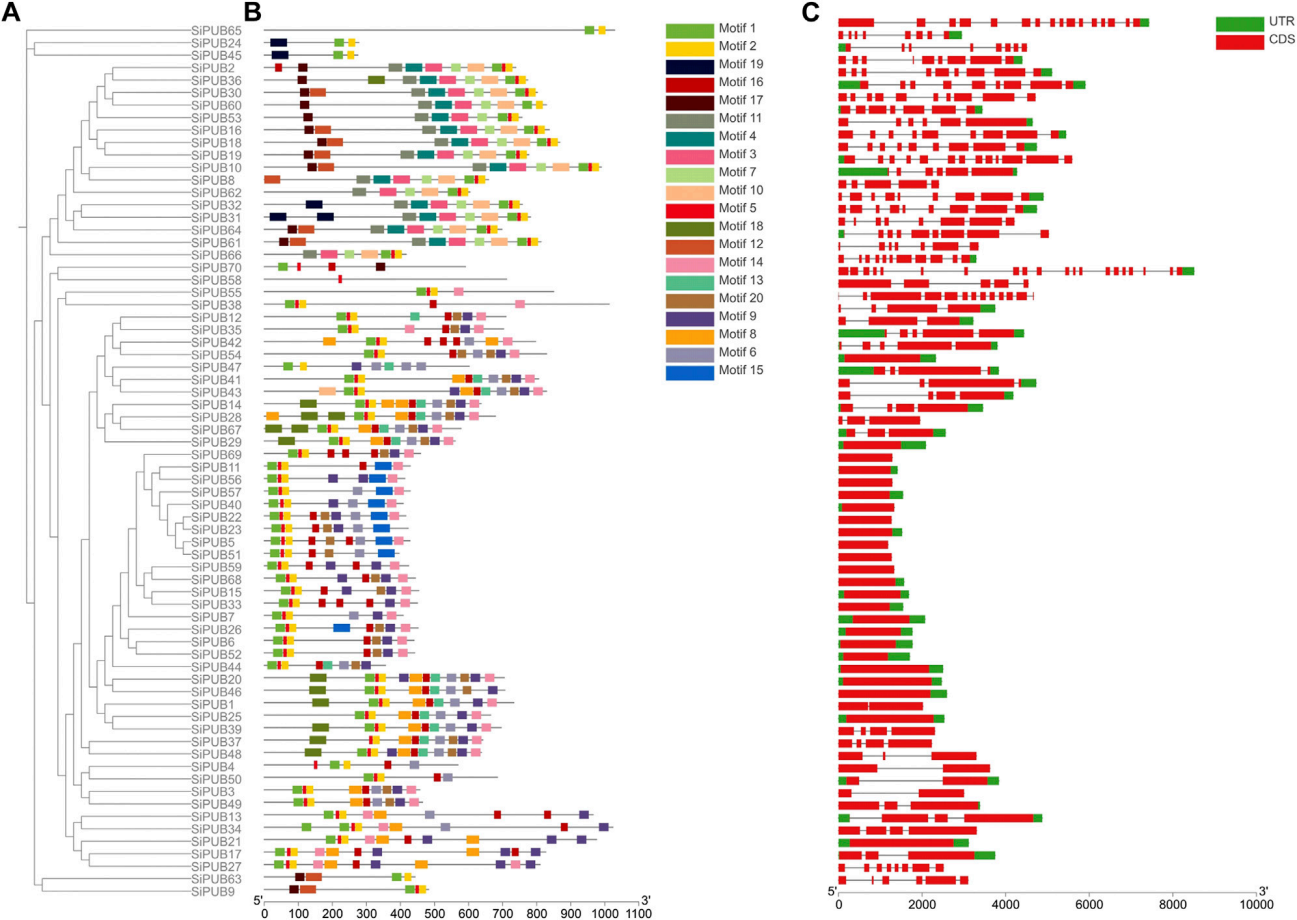
Gene structure and conserved motif analysis of *SiPUBs*. **(A)** Phylogenetic tree of 70 *SiPUBs*. **(B)** Conserved motifs of *SiPUBs*, with different colored squares representing different motifs. **(C)** Analysis of *SiPUB* gene structure. The red squares represent the coding sequence (CDS), the green squares represent the untranslated region (UTR), and the black lines between the two represent introns.

To further understand the possible structural evolutionary relationships of *SiPUBs*, we analyzed the phylogenetic tree, conserved motifs, and gene structure of *SiPUBs*. As shown in Figure 3, each family member had a different number of motifs, and members in the same subfamily generally had similar conserved motifs. The genes in subfamily I contained both motif 1 and motif 2 and some other motifs, while Subfamily II (*SiPUB65*) only contained motif 1 and motif 2. Subfamily III (*SiPUB24* and *SiPUB45*) contained only motif 1, 2, and 19. The conserved motifs in subfamily IV included motif 1, 2, 3, 7, 10, and 11. *SiPUB58* in subfamily V contained only motif 5, while *SiPUB38* contained motif 1, 2, 5, 14, and 16. Most members in subfamily VI contained motif 1, 2, 6, 8, 9, 13, 14, 16, and 18. Moreover, a total of 68 genes contained both motif 1 and motif 2, and 69 genes contained motif 1.

To further explore the diversity of *SiPUB* gene structure, we analyzed the intron-exon structure of *SiPUBs*. As shown in Figure 3, members in the same subfamily had similar intron arrangement, and most genes were broken genes, with the number of introns ranging from 0 to 19, and 24 genes having no introns. The structure of subfamily I genes was relatively simple, with most of members containing no introns, and only a few members contained 2–4 introns. Members in subfamily IV had 4–12 introns, with most genes containing eight introns. The genes in subfamily V had the largest number of introns, such as *SiPUB58* with 19 introns and *SiPUB38* with 12 introns. The introns in members from subfamily VI ranged from 0 to 3, with most genes containing 0 or 3 introns.

### 3.6 Promoter analysis and functional enrichment of SiPUBs

**FIGURE 4:**
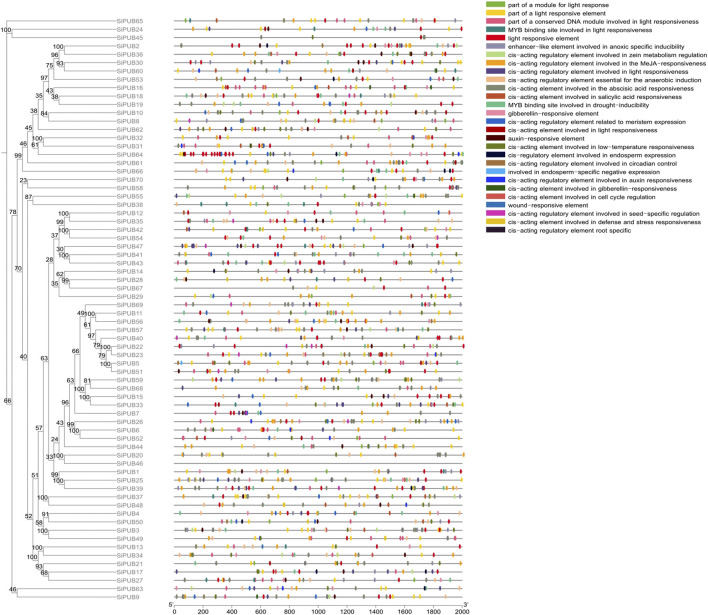
*SiPUB* promoter analysis. Rootless trees were generated by the neighbor-joining (NJ) method using MEGA11 software. Numbers next to branches indicate 1,000 bootstrap replicates as a percentage. Different colored squares represent different promoters. Detailed comments are included in the top right panel.

In order to better understand the mechanisms of gene regulation, we identified the *cis*-acting elements on each gene that can be used to study different environmental stress responses and tissue specificity. Finally, we identified 28 types of *cis*-acting elements, including light-responsive elements, plant hormone-responsive elements, abiotic stress-responsive elements, tissue-specific cell cycle elements and circadian rhythm control elements. *SiPUBs* showed differences in the type and number of *cis*-acting elements, among which *SiPUB23* had the most types of *cis*-acting elements (16 types), followed by *SiPUB18*, *SiPUB53,* and *SiPUB64* (15 types), and *SiPUB61* had the largest number of *cis*-acting elements (51), followed by *SiPUB25* (49). Sixty-nine genes contained light-responsive elements, while 68 genes had plant hormone-responsive elements, and it can be speculated that plant hormones may regulate these genes. The predicted stress-responsive elements mainly included those involved in anaerobic induction regulation, low temperature response, hypoxia induction, drought response, and trauma response. Among them, *cis*-acting elements involved in anaerobic induction regulation accounted for the largest proportion (53 genes), while trauma response elements were only found in *SiPUB13*, *SiPUB18*, and *SiPUB44*. There were 11 genes involved in endosperm specific negative expression; only 13 genes were involved in seed-specific regulation; while 37 genes were involved in molecular tissue-specific activation and expression (Figure 4). These results suggested that *SiPUBs* may be involved in foxtail millet’s response to biotic and abiotic stresses as well as tissue-specific responses.

**FIGURE 5:**
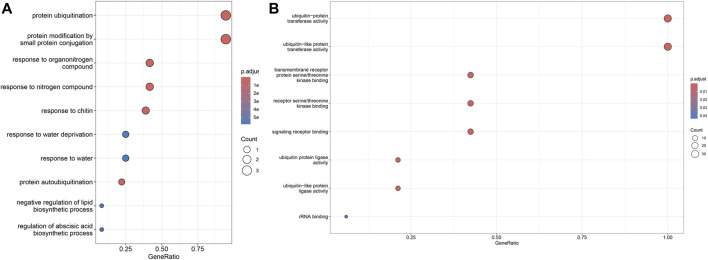
GO enrichment analysis. The figure shows top ten enrichment pathways. **(A)** Enrichment pathway of *SiPUBs* in biological processes. **(B)** Enrichment pathway of *SiPUBs* in molecular function.

GO enrichment analysis showed that 36 of the 70 *SiPUBs* were classified as “biological processes” and 33 were classified as “molecular functions.” Among the “biological processes,” 34 genes were enriched in protein ubiquitination and protein modification through small protein pathways, respectively. Furthermore, 33 genes were enriched in the “molecular function” pathway of ubiquitin-protein transferase activity and ubiquitin-like protein transferase activity (Figure 5 and Supplementary Table S3).

### 3.7 Collinearity analysis of SiPUBs

**FIGURE 6:**
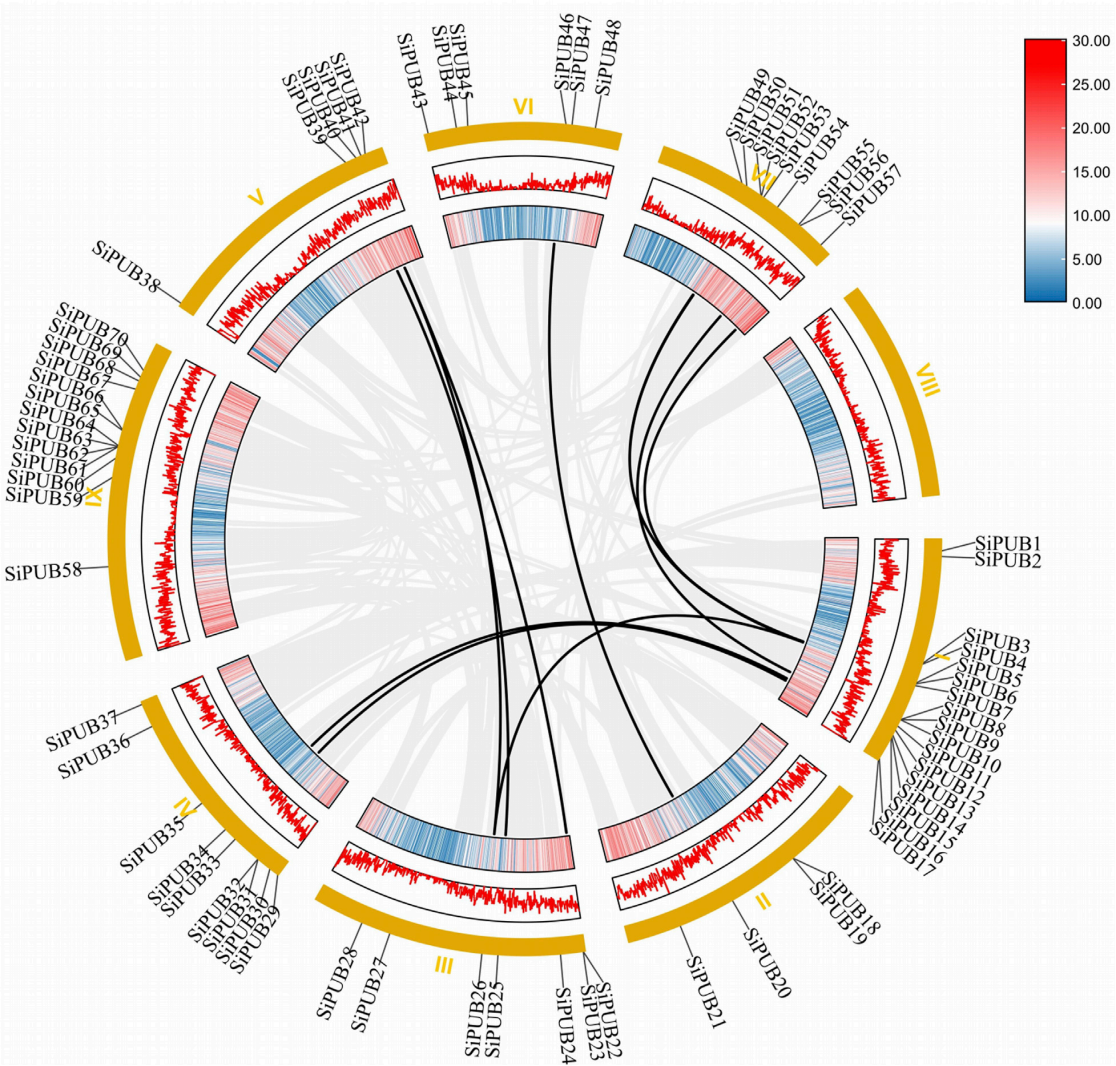
Collinearity analysis of *SiPUBs.* Chromosomes are shown on the outside, with yellow representing different chromosomes. The different colors of the inner circle represent the gene density of the chromosome (the gene density increases from blue to red), and the black line represents the fragment of repeating gene pairs of the foxtail millet.

To further understand the expansion of *SiPUB* family, we analyzed the fragment duplication gene pairs of foxtail millet. A total of 11 duplicate gene pairs of *U-box* genes were identified on the foxtail millet chromosome (*SiPUB6* and *SiPUB26*, *SiPUB13* and *SiPUB34*, *SiPUB15* and *SiPUB33*, *SiPUB5* and *SiPUB51*, *SiPUB6* and *SiPUB52*, *SiPUB11* and *SiPUB56*, *SiPUB5* and *SiPUB57*, *SiPUB20* and *SiPUB46*, *SiPUB25* and *SiPUB39*, *SiPUB26* and *SiPUB40*, *SiPUB22* and *SiPUB40*) (Figure 6). Among different chromosomes, chromosome I contained the most pairs of genes and the highest collinearity. Ks, Ka, and Ka/Ks of 11 *U-box* duplicate gene pairs in foxtail millet were calculated and analyzed (Table 2). The results showed that the Ka/Ks ratio of four duplicate gene pairs was much lower than 1, and the remaining seven duplicate gene pairs had Ka/Ks ratio close to 1. It can be speculated that gene duplication events may have contributed to the evolution of the *SiPUB* family.

**TABLE 2:** Calculation of Ka/Ks for *SiPUBs* duplicate gene pairs.

Duplicated gene pairs	Synonymous mutation rate (Ks)	Non-synonymous mutation rate (Ka)	Ka/Ks	Duplicated	Selection
*SiPUB6/SiPUB26*	0.55	0.73	0.76	Segmental	Yes
*SiPUB6/SiPUB52*	0.18	0.48	0.38	Segmental	Yes
*SiPUB26/SiPUB40*	0.68	0.80	0.85	Segmental	Yes
*SiPUB13/SiPUB34*	0.23	0.97	0.24	Segmental	Yes
*SiPUB15/SiPUB33*	0.23	0.56	0.42	Segmental	Yes
*SiPUB11/SiPUB56*	0.19	0.42	0.46	Segmental	Yes
*SiPUB20/SiPUB46*	0.25	0.47	0.54	Segmental	Yes
*SiPUB25/SiPUB39*	0.16	0.42	0.38	Segmental	Yes
*SiPUB22/SiPUB40*	0.41	0.61	0.67	Segmental	Yes
*SiPUB5/SiPUB51*	0.24	0.54	0.44	Segmental	Yes
*SiPUB5/SiPUB57*	0.59	0.83	0.71	Segmental	Yes

**FIGURE 7:**
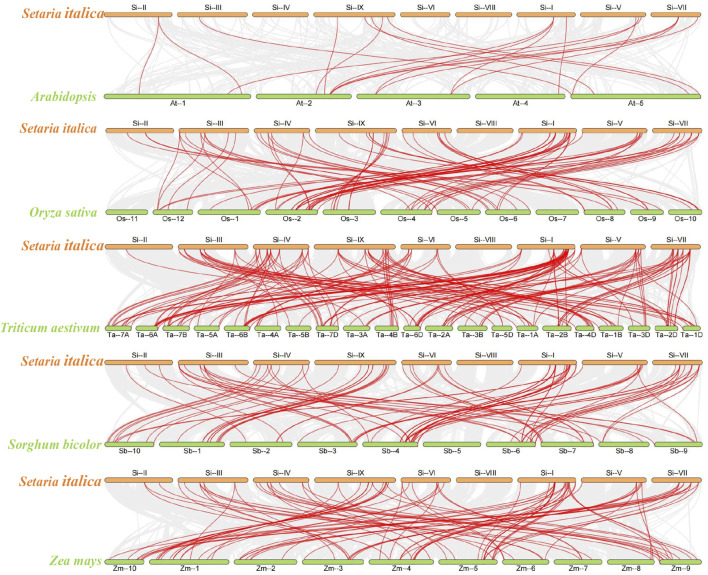
Analysis of U-box collinearity between foxtail millet and *Arabidopsis*, rice, sorghum, wheat, and maize. The gray line represents collinearity between all genes across species, and the red line represents collinearity between U-box genes across species.

In order to further elucidate the evolutionary relationship of *SiPUBs*, we constructed collinearity maps of *U-box* genes from foxtail millet and *Arabidopsis*, rice, and sorghum. (Figure 7). The results showed that 19 pairs of *U-box* genes had collinearity between foxtail millet and *Arabidopsis*, among which chromosome 2 in *Arabidopsis* had the most collinear genes. Moreover, 78 pairs of *U-box* genes were collinear between foxtail millet and rice, among which chromosome I of foxtail millet and chromosome 2 of rice had the most collinear genes. The *U-box* genes had the highest collinearity between foxtail millet and wheat (164 pairs), and chromosome I of foxtail millet and chromosome 7D of wheat had the most collinear genes. There were 85 pairs of *U-box* genes with collinearity between foxtail millet and sorghum, among which chromosome I of foxtail millet and chromosome 4 of sorghum had the most collinear genes. Collinearity of *U-box* genes was not found on chromosome 4 of *Arabidopsis*, chromosome 11, chromosome 7 of rice, chromosome 5 of sorghum.

### 3.8 Protein network interaction prediction in SiPUBs

**FIGURE 8:**
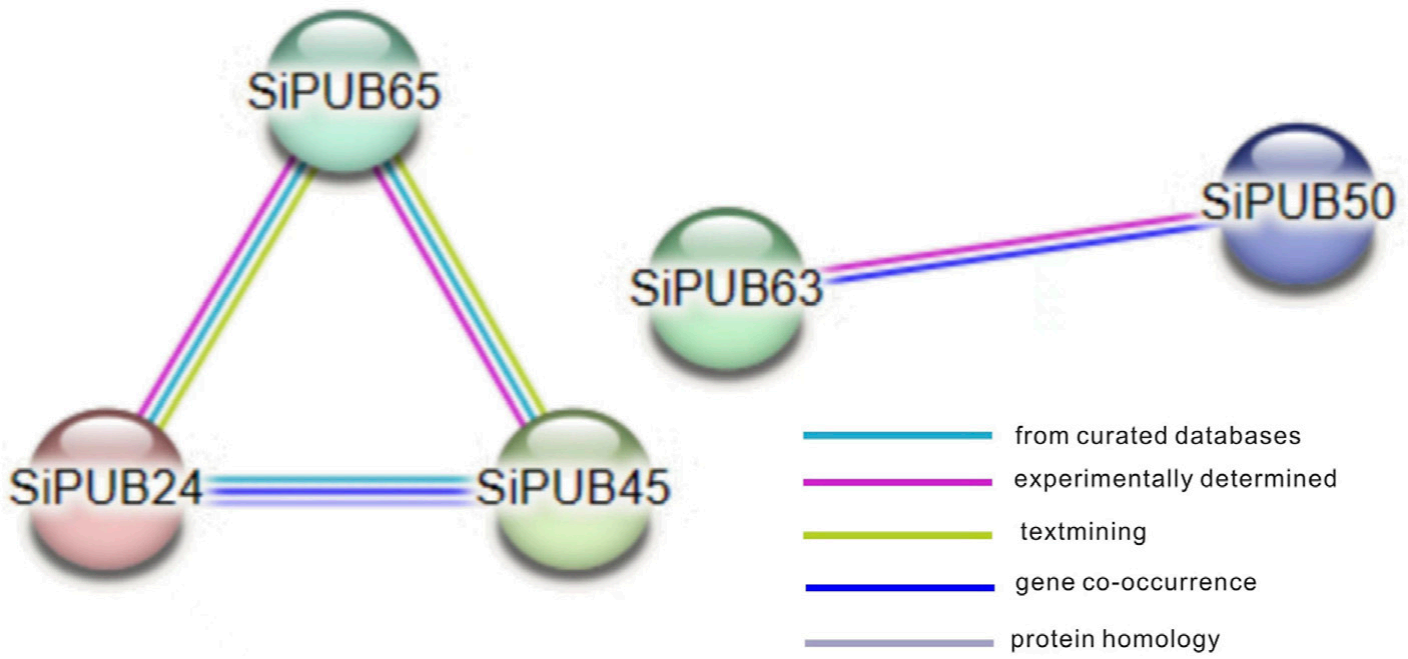
Protein network interaction prediction in SiPUBs.

To better understand the possible interactions between SiPUBs, the interaction between SiPUBs was predicted online using the STRING website. As shown in Figure 8, there were certain interactions among SiPUBs, including paired interactions among *SiPUB65*, *SiPUB24*, and *SiPUB45*, and interaction between *SiPUB63* and *SiPUB50*.

### 3.9 Expression patterns of SiPUBs in different tissues

**FIGURE 9:**
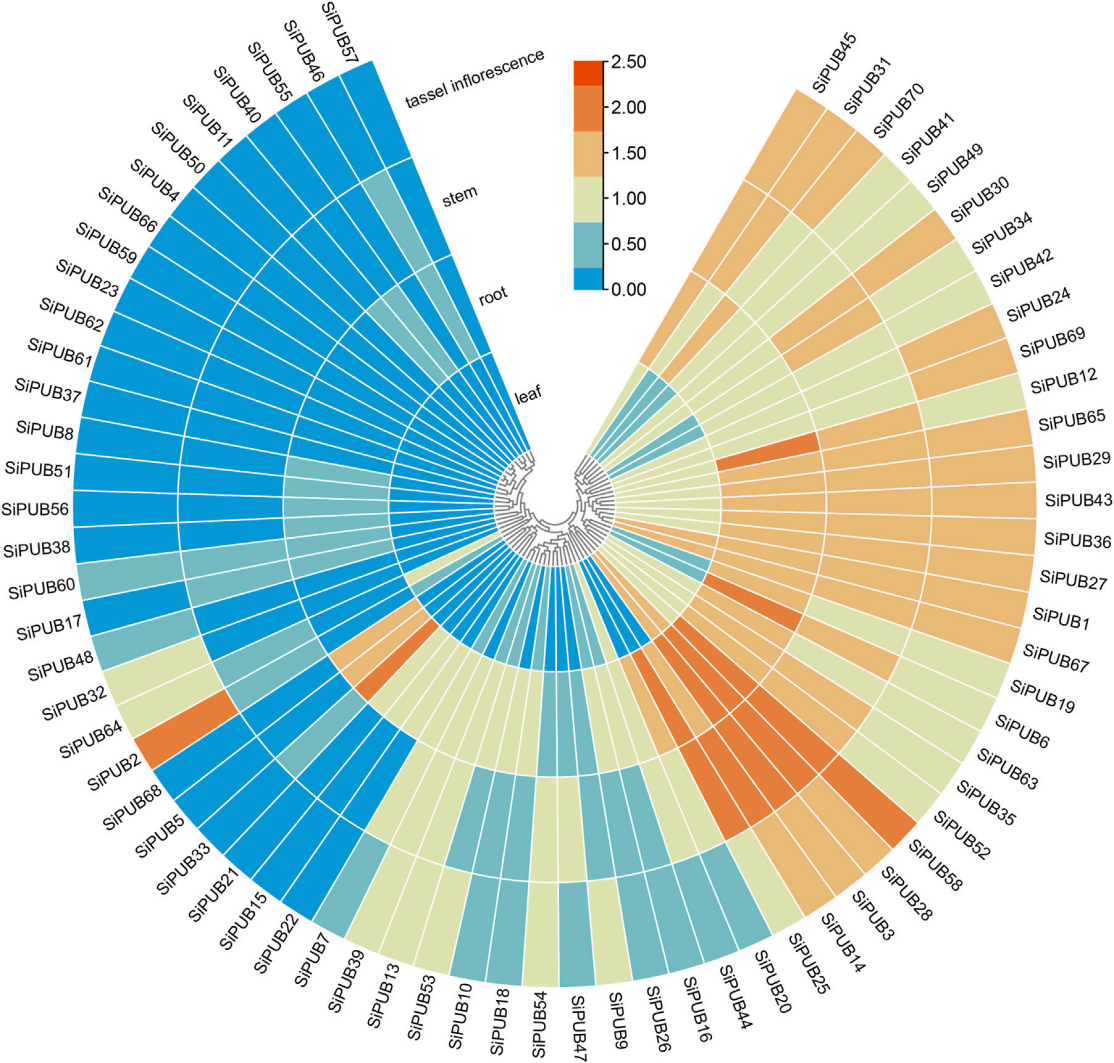
Heatmap of tissue-specific expression of *SiPUBs.* The color bar represents the log2 expression level of each gene (FPKM, fragments per kilobase of exon per million fragments mapped). Color bar annotation is included at the top of the image. The heatmap is colored according to expression values, with blue, yellow, and red representing at low, medium, and high transcription abundance, respectively.

Based on plant databases, the transcription levels of *SiPUBs* in different tissues (roots, stems, leaves, and inflorescences) were analyzed. Differences in the expression of *SiPUBs* were found among different tissues (Figure 9). Among them, *SiPUB1/3/28/67* were highly expressed in leaves. SiPUB3/6/12/25/28/33/58 were highly expressed in roots. SiPUB3/14/25/28/58 were highly expressed in the stem and SiPUB2/58 had high expression in the inflorescence. The relative expression patterns of *SiPUBs* in different tissues predicted their complex role in foxtail millet growth and development.

### 3.10 Responses of SiPUBs to saline-alkali stress detected by qRT-PCR

**FIGURE 10:**
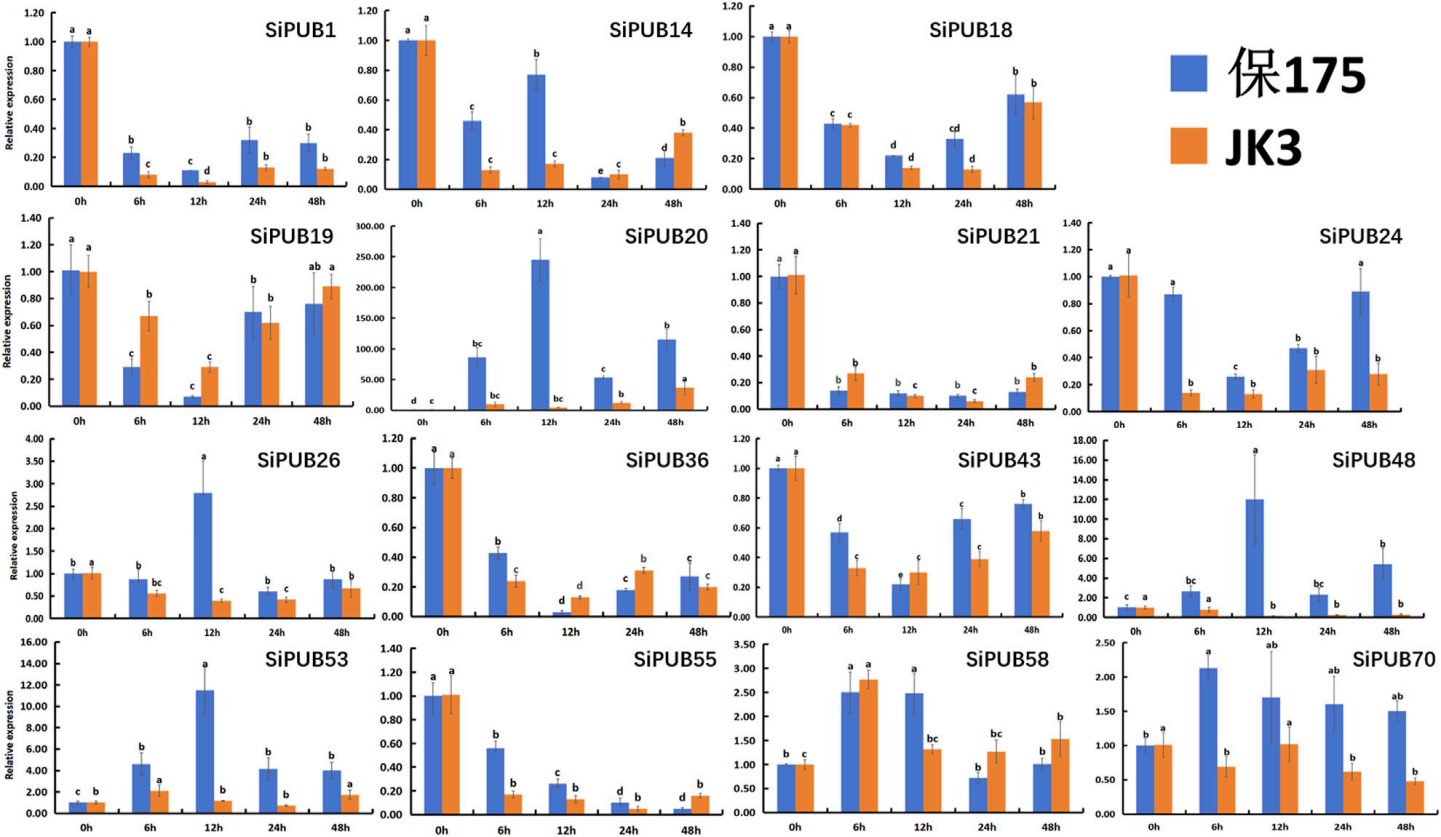
Expression patterns of some *SiPUBs* under saline-alkali treatment.

The results of tissue expression patterns showed that *SiPUBs* were expressed in all tissues of foxtail millet, and most of the genes were expressed or highly expressed in leaves. Leaves play a very important role in plants and are the main organs for photosynthesis, gas exchange, and transpiration. Therefore, they can most directly reflect the growth state of plants under abiotic stress. At present, the roles of U-box gene family members in foxtail millet have not been reported in detail, and the gene expression level of *SiPUBs* under salt-alkali stress remains clear. In order to explore the key role of *SiPUBs* in salt-alkali tolerance, 15 *SiPUBs* in leaf tissues under saline-alkali stress were randomly selected for qRT-PCR detection. The results showed that there were significant changes in the expression levels of these *SiPUBs* in B175 (sensitive) and JK3 (tolerant) under saline-alkali stress (Figure 10). In B175, the relative expression of *SiPUB1/18/19/21/24/36/43* first decreased and then increased with the extension of treatment time. The relative expression of *SiPUB14* firstly decreased, then increased, then decreased, and finally increased. The relative expression levels of *SiPUB20/48/58* first increased, then decreased, and finally increased, and became significantly higher than the initial levels at 48 h. The relative expression of *SiPUB26/53/70* first increased and then decreased, and the relative expression of *SiPUB53/70* at 48 h was significantly higher than the initial level. The relative expression of *SiPUB55* showed a consistent decreasing trend. For JK3, along with the extension of treatment time, the relative expression of *SiPUB1/14/18/19/21/24/26/43/55* showed a first declining and then rising trend; that of *SiPUB20* showed a consistently increasing trend, and became significantly higher than the initial level at 48 h. The relative expression of *SiPUB36/70* first decreased, then increased, and finally decreased. The relative expression of *SiPUB48* always showed a decreasing trend. The relative expression levels of *SiPUB53/58* first increased, then decreased, and finally increased, and became higher than the initial level at 48 h. The changes in the relative expression levels of *SiPUB1/18/19/21/24/43* (decreasing and then increasing) and *SiPUB58* (increasing, decreasing, and then increasing) were consistent between the two varieties, but there were significant differences in the relative expression levels. In addition, the relative expression level of *SiPUB20* in the two varieties was upregulated compared with that in the control, and was higher in B175 than in JK3 at different treatment time. For example, its expression level in B175 was 57.81 times that in JK3 at 12 h. Compared with that of the control, the relative expression of *SiPUB48/70* was upregulated in B175, but downregulated in JK3. The relative expression of *SiPUB48* in B175 was 92.23 times higher than that in JK3 at 12 h. At 6 h, the relative expression of *SiPUB70* in B175 was 3.09 times that in JK3. With the extension of saline-alkali treatment, the relative expression of *SiPUB26* was only upregulated in B175 at 12 h, which was 6.98 times that of JK3, but was downregulated in the two varieties at all other time points.

### 3.11 Protein structure analysis of SiPUBs

**FIGURE 11:**
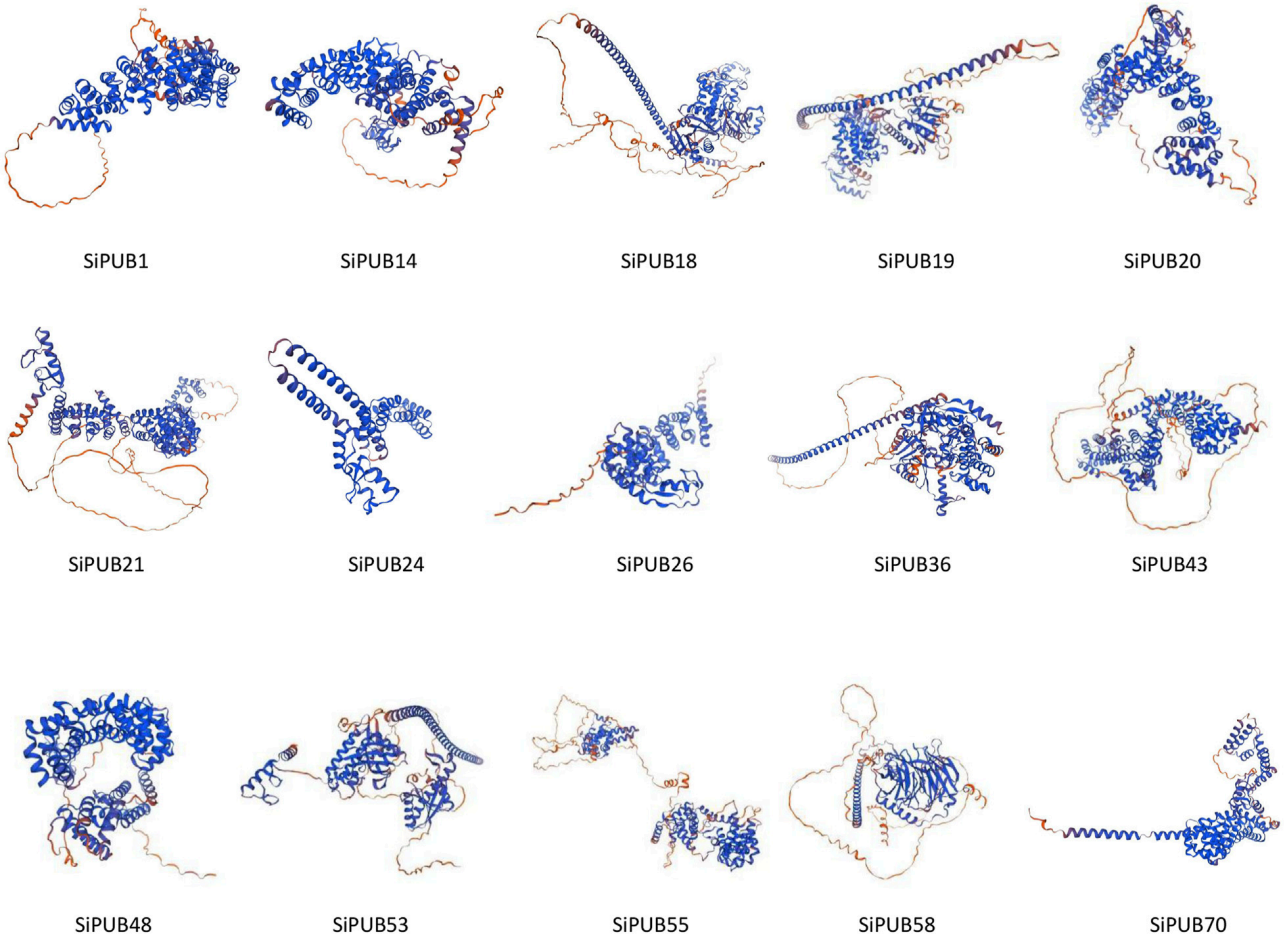
Prediction of protein tertiary structure in some *SiPUBs*.

The prediction of the tertiary structure of the protein showed that the coverage of the target sequence and the model sequence was 73% or more, and the conservation of SiPUBs was not strong, and there were obvious differences among all proteins (Figure 11). The consistency of *SiPUB21* and *SiPUB58* genes with the model protein reached 100%, and the tertiary structure was predicted to have a generally good consistency with the model protein.

## 4 Discussion

### 4.1 SiPUBs are conserved during evolution

Plant *U-box* proteins constitute a small protein family with a *U-box* domain (Azevedo et al., 2001; Wiborg et al., 2008; Chen and Hellmann, 2013). The *U-box* domain consists of approximately 70 amino acids and maintains its conformation through interactions with components such as hydrogen bonds and salt bridges (Aravind and Koonin, 2000; Andersen et al., 2004). *U-box* E3 is involved in a variety of biological processes, such as development, self-incompatibility, and response to hormones, and are widely associated with plant stress response (Yee and Goring, 2009; Lee and Kim, 2011; Lyzenga and Stone, 2012; Chen and Hellmann, 2013; Duplan and Rivas, 2014; Stone, 2014). *U-box* genes play important roles in response to drought (Cho et al., 2006; Cho et al., 2008; Liu et al., 2011; Seo et al., 2012), salt (Cho et al., 2006; Ni et al., 2010; Bergler and Hoth, 2011), temperature stress (Stone et al., 2003; Cho et al., 2006), oxidative stress (Park et al., 2011), and low phosphate stress (Hur et al., 2012). At present, *U-box* genes have been proved to have great biological significance in a variety of plants such as *Arabidopsis* (Lu et al., 2008). However, there has been no report on the *U-box* genes in foxtail millet. In this study, *SiPUBs* were systematically analyzed through bioinformatics analysis, and a total of 70 members were identified. The molecular weights of SiPUBs ranged from 30946.21 Da to 115391.56 Da, and the pI ranged from 5.17 to 8.98. In addition to the *U-box* domain, SiPUBs also contain the ARM, WD40 and TPR secondary domains, which are mainly used to mediate the specific recognition of *U-box* protein and substrate protein and maintain the basic function of the family, enriching the diversity of genes (Lu et al., 2020). Gene structure analysis also revealed that most *SiPUBs* contain multiple introns, indicating changes in their structure during evolution, and differences in intron/exon structure can often promote the evolution of multi-gene families, which may lead to different functions of members within the same subfamily (Rogozin et al., 2003). Similar results have also been obtained for the *U-box* gene families of other species such as cotton (Lu et al., 2020), banana (Hu et al., 2018), and sorghum (Fang et al., 2022). The number of exons varied greatly from 1 to 20, which may be attributed to the directed evolution of function and structure of *U-box* genes throughout the long evolutionary history. Furthermore, gene structure and phylogenetic analysis indicated that *U-box* genes in different branches of the same subfamily have different numbers of exons, introns, and protein sequence lengths. However, orthologous genes in the same branch showed highly similar physicochemical properties and gene structure. These results indicated that *SiPUBs* are highly conserved during evolution, but at the same time, the gene functions were diversified for adaptation to environmental changes and population continuation.

### 4.2 Tandem duplication events are the main driving force of SiPUB evolution

Gene duplication is the primary force driving the expansion of gene families over a period of time under the regulation of environmental and biological factors. Gene duplication is a universal phenomenon in plants, including tandem duplication, fragment duplication, and whole genome duplication (Kondrashov et al., 2002; Conant and Wolfe, 2008). Chromosomal localization analysis and collinearity analysis showed that gene duplication is the main cause of *SiPUB* diversity. Among the 70 *SiPUBs*, 12 were generated by tandem duplication, which is one of the main reasons for the expansion of this gene family. A total of 11 duplication *SiPUB* gene pairs were identified on foxtail millet chromosomes. Similar results were obtained in other species as well. In tomato, the *U-box* gene family was found to experience ten duplication events (Sharma and Taganna, 2020). In cotton, 19 *U-box* genes were derived from tandem duplication (Lu et al., 2020). In cabbage, there are 24 pairs of tandem duplicated *U-box* genes (Hu et al., 2019). These results indicate that subfunctionalization or neofunctionalization of duplicate genes can lead to the expansion of gene families as well as structural organization and diversification of gene expression. Over time, the preserved sequences may undergo new functionalization to produce differentiation.

### 4.3 Different subfamilies of SiPUBs have differential functions

The conserved U-box domain of *SiPUBs* is often associated with ARM repeats, WD40 repeats, TRR structures, and other domains. *SiPUBs* can be divided into six subfamilies according to phylogenetic relationships and domain composition. Due to the conserved nature of genes, genes with similar or identical functions are located in the same subfamily, which lays a solid foundation for studying the functions of *SiPUBs*.

There is only one UFD2 gene in foxtail millet and *Arabidopsis* (AtUFD2). AtUFD2 protein contains a conserved domain similar to yeast UFD2 protein, which can interact with aaa ATPase CDC48 and participates in the regulation of cell cycle, death, organelle formation, and other physiological activities (Zhou et al., 2021). In *Arabidopsis*, AtCHIP in the TPR + *U-box* subfamily is involved in regulating abiotic stress response. Under low temperature and dark conditions, AtCHIP alters the activity of PP2A in stress response signal transduction, thus responding to abiotic stress (Luo et al., 2006). The *U-box* + ARM subfamily is the most extensively studied subfamily. *AtPUB18*, *AtPUB19*, *AtPUB46*, and *AtPUB48*, which belong to the *U-box* + ARM subfamily, play important roles in drought stress response (Bergler and Hoth, 2011; Adler et al., 2017). AtPUB18 and AtPUB19 are conserved proteins containing PUB-ARM, a type of E3 ubiquitin ligase. During the early development of *Arabidopsis*, *AtPUB18* and *AtPUB19* inhibit seed germination under salt stress, and the salt stress response would disappear in the absence of PUB-ARM proteins, indicating that ubiquitination is a key element stimulating salt sensitivity (Bergler and Hoth, 2011). *AtPUB17* positively regulates the processes of cell apoptosis and plant defense (Sadanandom, 2007), while *AtPUB9* regulates lateral root development under phosphate starvation conditions (Sankaranarayanan and Samuel, 2015). On this basis, it can be inferred that other genes in the *U-box* + ARM subfamily may have similar or overlapping functions. The *U-box* only subfamily (subfamily I in this study) has a simple gene structure and plays important roles in plant growth, development, and abiotic stress response. Previous studies have shown that apple callus overexpressing *MdPUB24* and *Arabidopsis* seedlings with ectopic expression of *MdPUB24* exhibited significant decreases in growth compared with the wild type under salt stress conditions, indicating that *MdPUB24* negatively regulates salt stress response (Qi et al., 2017). Based on the homology of genes in the evolutionary relationship, it can be speculated that homologous genes of *Arabidopsis* and foxtail millet in the same subfamily may also participate in similar regulatory pathways.

### 4.4 Potential function and expression analysis of SiPUBs

Protein ubiquitination is a post-translational modification involved in various physiological processes in plants and is required for many signaling pathways in many important cellular processes such as plant growth, development and eukaryotic stress response (You et al., 2016; Ye et al., 2018). Ma et al., 2021 showed that MYB6 and VDAL are effector factors with the same regulatory effect on verticillium wilt resistance; *PUB25* and *PUB26* can degrade MYB6 through ubiquitination; and VDAL competes with MYB6 to bind *PUB25* and *PUB26* to promote resistance. Previous studies have also found that *PUB25* and *PUB26* have two forms of ubiquitination modification of MYB15 protein at low temperatures. At the early stages of cold stress, *PUB25* and *PUB26* promote the degradation of MYB15 via high-level K48 linkages and stabilize ICE1 by promoting its K63-linked ubiquitination, thereby facilitating the inhibition of MYB15 by ICE1 and leading to activation of CBF expression. Upon prolonged cold stress, *PUB25* and *PUB26* enhance K48-linked modification on ICE1 to target it to the 26S proteasome for degradation while simultaneously adding K63 linkages to MYB15, allowing it to bind CBF promoters and repress their expression (Wang et al., 2023). The E3 ubiquitin ligases *PUB12* and *PUB13* directly interact with BRI1 and ubiquitinate BRI1, and *PUB12/PUB13-*mediated ubiquitination regulates endocytosis and intracellular degradation of BRI1 (Zhou et al., 2018). However, there have been few reports on the function of *U-box* (*PUB*) gene in foxtail millet, but it can be inferred from previous studies that *U-box* gene family in foxtail millet may have similar functions.

Promoter analysis can effectively predict the gene family associations in stress-related mechanism, hormone regulation, and development (Sharma and Taganna, 2020). Similar patterns were observed in the promoter analysis of purple clover by Yang et al. (2017), indicating a common association of the *U-box* gene family with plant stress and hormone regulatory pathways. Similar to previous studies, this study conducted promoter function prediction analysis on *SiPUBs* and found that most *SiPUBs* contain a large number of light-responsive elements, indicating that *SiPUBs* may be involved in the regulation of plant photosynthesis. Moreover, a large number of plant hormone-responsive elements such as auxin, abscisic acid, and gibberellin were found in most of the genes, suggesting that *SiPUBs* may be involved in a series of other processes such as the growth and development of foxtail millet plants and the ripening of fruit ears.

Previous studies have demonstrated that *Arabidopsis PUB4* mutant exhibited higher levels of cell proliferation and division in the root and shoot apical meristem (Kinoshita et al., 2015). In this study, expression analysis of *SiPUBs* in different tissues showed that *SiPUBs* had obvious tissue specificity, most of which had the relatively high expression in roots. Similarly, *U-box* genes also showed obvious tissue specificity in banana, with high expression levels in the root (Hu et al., 2018). This pattern of expression has also been found in other plants such as soybean (Wang et al., 2020a) and sorghum (Fang et al., 2022). There results suggest that *U-box* genes may affect root development or other growth and development processes of plants.

As a matter of fact, previous studies have demonstrated that *U-box* genes are involved in plant growth and development. For example, *TaPUB4* has been found to participate in the regulation of pollen development by modulating the metabolism of sucrose and starch in the anther (Zhang et al., 2017). Since the expression levels of genes in different tissues and cell types can reveal their functions in organisms and roles in different physiological processes, we conducted qRT-PCR analysis on 15 *SiPUBs* under saline-alkali stress in this study. The results showed that all 15 genes were induced to express under saline-alkali stress, and the relative expression levels of some genes were significantly different between B175 and JK3. In B175, the expression level of *SiPUB20* from subfamily VI was more significantly induced under salt and alkali stress than that of other genes, and its expression level was 53.34–245.1 folds upregulated compared with the control, and the expression level varied greatly at different treatment time. The relative expression level of *SiPUB20* in JK3 was 4.20–36.83 folds higher than that in B175 at different treatment time. The expression level of *SiPUB20* was the most different between JK3 and B175, and the relative expression level of B175 was 57.81 folds that of JK3 after 12 h of treatment. The relative expression levels of both *SiPUB48* in subfamily VI and *SiPUB70* in subfamily I were upregulated in B175 while downregulated in JK3. Previous studies have found that *TaPUB1* in wheat can enhance plant salt tolerance and drought resistance. Additionally, *TaPUB15* is induced by salt, abscisic acid, low temperature, and polyethylene glycol (PEG) treatment (Zhang et al., 2017). Hwang et al. (2014) demonstrated that inhibition of *AtPUB30* in *Arabidopsis* can enhance salt tolerance during germination. Cho et al. (2006) found that *CaPUB1* from pepper could increase plant salt tolerance when overexpressed in *Arabidopsis*. Han et al. (2019) indicated that *MdPUB29* in apple may positively regulate salt tolerance. Further analysis in this study showed that the expression levels of nine out of 15 *SiPUB* genes in the two varieties were lower than those of the control under saline-alkali treatment, and the downregulation degree in saline-alkali tolerant variety JK3 was higher than that in saline-alkali sensitive variety B175. Previous studies have found that *Arabidopsis* transgenic plants overexpressing *pub22* and *pub23* are sensitive to drought stress, and *pub22* and *pub23* functionally deficient mutant plants are significantly more drought-tolerant, and *pub22* and *pub23* double mutant plants are even more drought-tolerant. These results indicated that *PUB22* and *PUB23* play a negative regulatory role in water stress response (Cho et al., 2008). Similar to these previous studies, this study suggested that *U-box* genes may play a critical role in the response against saline-alkali stress through negative regulation. Furthermore, subcellular localization analysis predicted that *SiPUB20* is localized on membrane-bound organelles or plasma membrane, indicating that it may be involved in saline-alkali response by changing membrane composition or ion transport. Moreover, the localization of *SiPUB48/70* in the nucleus suggests their potential involvement in transcriptional regulation or nucleus transport processes in response to saline-alkali stress.

## 5 Conclusion

In this study, a total of 70 *SiPUBs* were identified, all of which contained the *U-box* conserved domain. The 70 *SiPUBs* were distributed on eight chromosomes of foxtail millet, forming five tandem gene clusters, and could be divided into six subfamilies based on the phylogenetic relationships and domain composition. *SiPUBs* contained a total of 28 types of *cis*-acting elements, including light-responsive elements, plant hormone-responsive elements, abiotic stress-responsive elements, tissue-specific cell cycle, and circadian rhythm control, suggesting that *SiPUBs* may be involved in abiotic stress responses. Most *SiPUBs* showed high expression in roots, stems, leaves, and flowers, suggesting their crucial roles in plant development. Saline-alkali stress resulted in significantly differential expression of 15 randomly selected *SiPUBs*. Gene expression pattern analysis indicted that *SiPUB20/48/70* may play important roles in the response to saline-alkali stress. The results of this study lay a foundation for further exploring the mechanism for the response of *SiPUBs* to saline-alkali stress, and provide reference for molecular breeding of saline-alkali tolerance in foxtail millet.

## Data Availability

The original contributions presented in the study are included in the article/Supplementary Material, further inquiries can be directed to the corresponding authors.
