# Supplementary material for: Genome-wide identification of U-box gene family and expression analysis in response to saline-alkali stress in foxtail millet (Setaria italica L. Beauv)
Source: Front Genet. 2024 Feb 16;15:1356807. doi: 10.3389/fgene.2024.1356807 (PMC10904469; doi:10.3389/fgene.2024.1356807)
Supplement: Supplementary file 1 [file Table1.DOCX]

**Table S1.** Basic information of *SiPUBs*.

| **Gene** | **Gene-id** | **chr** | **Number of Amino Acid** | **Molecular Weight (Da)** | **Theoretical pI** | **Instability Index** | **Aliphatic Index** | **Grand Average of Hydropathicity** | **details** |
| --- | --- | --- | --- | --- | --- | --- | --- | --- | --- |
| *SiPUB17* | SETIT_016314mg | Ⅰ | 827 | 89319.39 | 5.35 | 44.21 | 104.55 | -0.016 | Cytosol |
| *SiPUB1* | SETIT_016453mg | Ⅰ | 733 | 79286.64 | 8.92 | 50.91 | 102.16 | 0.019 | Plasma membrane |
| *SiPUB5* | SETIT_017274mg | Ⅰ | 428 | 46480.73 | 8.98 | 48.32 | 97.62 | 0.004 | Cytosol |
| *SiPUB2* | SETIT_016441mg | Ⅰ | 739 | 81922.36 | 6.04 | 49.9 | 86.73 | -0.371 | Chloroplast |
| *SiPUB12* | SETIT_016484mg | Ⅰ | 710 | 79839.89 | 5.47 | 48.01 | 85.3 | -0.261 | Chloroplast |
| *SiPUB10* | SETIT_016212mg | Ⅰ | 990 | 111796.06 | 5.69 | 48.69 | 81.15 | -0.52 | Nucleus |
| *SiPUB3* | SETIT_017157mg | Ⅰ | 457 | 49439.35 | 6.01 | 46 | 92.78 | -0.229 | Cytosol |
| *SiPUB11* | SETIT_020151mg | Ⅰ | 429 | 45853.14 | 5.4 | 44.37 | 105.1 | 0.214 | Vacular membrane |
| *SiPUB7* | SETIT_017372mg | Ⅰ | 408 | 42198.46 | 8.18 | 34.01 | 97.25 | 0.124 | Chloroplast |
| *SiPUB16* | SETIT_016305mg | Ⅰ | 837 | 90785.85 | 5.33 | 46.79 | 79.96 | -0.439 | Nucleus |
| *SiPUB6* | SETIT_017223mg | Ⅰ | 440 | 45200.08 | 8.69 | 53.41 | 95.41 | 0.186 | Chloroplast |
| *SiPUB13* | SETIT_016226mg | Ⅰ | 966 | 106991.62 | 6.08 | 44.68 | 101.57 | -0.074 | Chloroplast |
| *SiPUB9* | SETIT_017060mg | Ⅰ | 483 | 55402.83 | 5.96 | 49.4 | 83.62 | -0.617 | Cytosol |
| *SiPUB4* | SETIT_019301mg | Ⅰ | 569 | 62571.3 | 5.44 | 58.2 | 85.36 | -0.333 | Nucleus |
| *SiPUB15* | SETIT_017172mg | Ⅰ | 454 | 48534.06 | 8.93 | 49.37 | 97.84 | 0.049 | Chloroplast |
| *SiPUB14* | SETIT_016624mg | Ⅰ | 637 | 68944.83 | 5.94 | 44.74 | 101.49 | -0.143 | Chloroplast |
| *SiPUB8* | SETIT_016587mg | Ⅰ | 659 | 74174.19 | 6.14 | 53.88 | 79.7 | -0.487 | Chloroplast |
| *SiPUB18* | SETIT_028897mg | Ⅱ | 868 | 96731.5 | 5.98 | 47.73 | 79.46 | -0.496 | Chloroplast |
| *SiPUB20* | SETIT_029090mg | Ⅱ | 705 | 74354.8 | 8.24 | 44.36 | 104.18 | 0.167 | Plasma membrane |
| *SiPUB19* | SETIT_029006mg | Ⅱ | 777 | 87474.63 | 5.95 | 44.97 | 84.26 | -0.381 | Cytosol |
| *SiPUB21* | SETIT_032533mg | Ⅱ | 976 | 107624.9 | 8.74 | 43.64 | 98.65 | -0.148 | Nucleus |
| *SiPUB24* | SETIT_022917mg | Ⅲ | 278 | 31538.88 | 5.22 | 35.89 | 90.25 | -0.321 | Cytosol |
| *SiPUB25* | SETIT_024545mg | Ⅲ | 665 | 70330.85 | 7.02 | 48.61 | 103.79 | 0.091 | Chloroplast |
| *SiPUB27* | SETIT_021242mg | Ⅲ | 810 | 88974.92 | 5.5 | 37.67 | 106.53 | -0.063 | Cytosol |
| *SiPUB28* | SETIT_021390mg | Ⅲ | 679 | 73579.17 | 5.62 | 47.07 | 96.23 | -0.195 | Plasma membrane |
| *SiPUB26* | SETIT_022018mg | Ⅲ | 452 | 46180.56 | 8.86 | 49.39 | 101.11 | 0.25 | Chloroplast |
| *SiPUB22* | SETIT_022170mg | Ⅲ | 416 | 44837.82 | 8.23 | 47.93 | 102.07 | 0.103 | Endoplasmic reticulum |
| *SiPUB23* | SETIT_024634mg | Ⅲ | 423 | 45229.26 | 8.74 | 49.69 | 102.22 | 0.124 | Vacular membrane |
| *SiPUB37* | SETIT_008014mg | Ⅳ | 642 | 71204.43 | 5.98 | 45.85 | 106.28 | -0.182 | Chloroplast |
| *SiPUB34* | SETIT_005763mg | Ⅳ | 1024 | 111976.51 | 6.14 | 47.19 | 98.33 | -0.103 | Cytosol |
| *SiPUB32* | SETIT_005916mg | Ⅳ | 758 | 85705.5 | 6.02 | 50.01 | 85.62 | -0.462 | Cytosol |
| *SiPUB33* | SETIT_006416mg | Ⅳ | 450 | 46774.13 | 8.88 | 38.89 | 98.44 | 0.244 | Chloroplast |
| *SiPUB30* | SETIT_005883mg | Ⅳ | 802 | 89484.59 | 6.42 | 48.06 | 82.34 | -0.409 | Nucleus |
| *SiPUB29* | SETIT_006159mg | Ⅳ | 561 | 60407.57 | 6.62 | 37.72 | 99.25 | -0.14 | Cytosol |
| *SiPUB31* | SETIT_005894mg | Ⅳ | 782 | 88487.16 | 5.51 | 42.23 | 80.4 | -0.508 | Nucleus |
| *SiPUB36* | SETIT_005898mg | Ⅳ | 774 | 86283.88 | 7.57 | 53.17 | 92.84 | -0.265 | Cytosol |
| *SiPUB35* | SETIT_005963mg | Ⅳ | 703 | 78452.01 | 5.61 | 43.23 | 94.75 | -0.135 | Plasma membrane |
| *SiPUB62* | SETIT_039111mg | Ⅸ | 604 | 68589.55 | 5.55 | 48.18 | 84.49 | -0.548 | Cytosol |
| *SiPUB65* | SETIT_034048mg | Ⅸ | 1029 | 115391.56 | 5.17 | 47.65 | 95.71 | -0.141 | Chloroplast |
| *SiPUB59* | SETIT_035837mg | Ⅸ | 424 | 44507.57 | 6.36 | 43.83 | 111.56 | 0.317 | Vacular membrane |
| *SiPUB58* | SETIT_034460mg | Ⅸ | 712 | 77117 | 7.03 | 35.81 | 76.1 | -0.42 | Nucleus |
| *SiPUB64* | SETIT_034488mg | Ⅸ | 698 | 79151.88 | 6.72 | 48.98 | 88.71 | -0.296 | Nucleus |
| *SiPUB60* | SETIT_039355mg | Ⅸ | 829 | 91550 | 6.85 | 43.88 | 85.52 | -0.38 | Cytosol |
| *SiPUB69* | SETIT_035597mg | Ⅸ | 459 | 49266.57 | 7.49 | 42.17 | 105.08 | 0.131 | Cytosol |
| *SiPUB66* | SETIT_039907mg | Ⅸ | 417 | 47691.77 | 5.23 | 53.86 | 88.42 | -0.23 | Cytosol |
| *SiPUB61* | SETIT_034286mg | Ⅸ | 813 | 91245.7 | 7.01 | 47.78 | 80.12 | -0.459 | Chloroplast |
| *SiPUB63* | SETIT_035720mg | Ⅸ | 443 | 49977.87 | 5.57 | 56.24 | 81.51 | -0.448 | Cytosol |
| *SiPUB70* | SETIT_034816mg | Ⅸ | 591 | 65166.7 | 7.26 | 32.99 | 68.17 | -0.555 | Nucleus |
| *SiPUB67* | SETIT_034883mg | Ⅸ | 578 | 62443.84 | 5.25 | 42.43 | 95.12 | -0.241 | Chloroplast |
| *SiPUB68* | SETIT_035714mg | Ⅸ | 444 | 47283 | 8.6 | 42.51 | 105.16 | 0.148 | Chloroplast |
| *SiPUB40* | SETIT_001665mg | Ⅴ | 408 | 43165.06 | 8.91 | 55.83 | 109.36 | 0.207 | Endoplasmic reticulum |
| *SiPUB42* | SETIT_000355mg | Ⅴ | 797 | 87282.97 | 5.62 | 51.31 | 86.41 | -0.282 | Nucleus |
| *SiPUB41* | SETIT_000346mg | Ⅴ | 806 | 88124.06 | 5.22 | 45.28 | 97.15 | -0.2 | Chloroplast |
| *SiPUB39* | SETIT_000518mg | Ⅴ | 696 | 73872.82 | 6.15 | 50.29 | 98.72 | 0.027 | Chloroplast |
| *SiPUB38* | SETIT_000162mg | Ⅴ | 1013 | 110637.73 | 6.09 | 47.72 | 94.76 | -0.12 | Chloroplast |
| *SiPUB46* | SETIT_013335mg | Ⅵ | 707 | 73694.76 | 7.93 | 38.31 | 104.57 | 0.16 | Plasma membrane |
| *SiPUB44* | SETIT_013997mg | Ⅵ | 356 | 36948.61 | 6.96 | 47.74 | 108.71 | 0.169 | Cytosol |
| *SiPUB48* | SETIT_015737mg | Ⅵ | 638 | 70679.9 | 7.05 | 46.57 | 102.51 | -0.289 | Nucleus |
| *SiPUB47* | SETIT_013440mg | Ⅵ | 602 | 62213.37 | 7.11 | 68.16 | 82.77 | -0.135 | Chloroplast |
| *SiPUB45* | SETIT_014243mg | Ⅵ | 275 | 30946.21 | 6.04 | 38.21 | 85.96 | -0.477 | Mito chondrion |
| *SiPUB43* | SETIT_013258mg | Ⅵ | 829 | 89560.11 | 5.56 | 46.17 | 94.76 | -0.26 | Chloroplast |
| *SiPUB51* | SETIT_012592mg | Ⅶ | 396 | 42140.51 | 7.24 | 50.57 | 96.41 | -0.026 | Cytosol |
| *SiPUB54* | SETIT_009362mg | Ⅶ | 829 | 91565.53 | 5.73 | 47.61 | 94.64 | -0.22 | Golgi apparatus |
| *SiPUB49* | SETIT_012408mg | Ⅶ | 465 | 50561.68 | 5.94 | 54.79 | 89.91 | -0.24 | Cytosol |
| *SiPUB57* | SETIT_012788mg | Ⅶ | 429 | 44917.91 | 7.15 | 50.81 | 103.78 | 0.189 | Cytosol |
| *SiPUB50* | SETIT_012240mg | Ⅶ | 685 | 73927.77 | 5.7 | 59.26 | 80.69 | -0.313 | Nucleus |
| *SiPUB53* | SETIT_009444mg | Ⅶ | 757 | 85903.4 | 7.62 | 47.71 | 83.51 | -0.399 | Chloroplast |
| *SiPUB55* | SETIT_011652mg | Ⅶ | 850 | 91268.52 | 6.16 | 54.16 | 82.95 | -0.256 | Chloroplast |
| *SiPUB56* | SETIT_010229mg | Ⅶ | 413 | 44447.46 | 8.45 | 47.77 | 98.77 | 0.111 | Cytosol |
| *SiPUB52* | SETIT_010124mg | Ⅶ | 442 | 45437.06 | 6.49 | 43.34 | 95.18 | 0.161 | Chloroplast |
