# Supplementary material for: Genome-wide identification of U-box gene family and expression analysis in response to saline-alkali stress in foxtail millet (Setaria italica L. Beauv)
Source: Front Genet. 2024 Feb 16;15:1356807. doi: 10.3389/fgene.2024.1356807 (PMC10904469; doi:10.3389/fgene.2024.1356807)
Supplement: Supplementary file 3 [file Table2.DOCX]

**Table S2.** Secondary structure analysis of *SiPUBs*.

| **Gene** | **Alpha-helix (%)** | **Extended strand (%)** | **Beta-sheet (%)** | **Random coil (%)** | **Distribution of secondary structure elements** |
| --- | --- | --- | --- | --- | --- |
| *SiPUB17* | 62.1（514） | 4.47（37） | 4.96（41） | 28.42（235） | 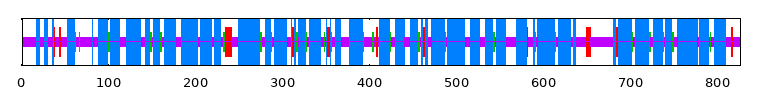 |
| *SiPUB1* | 53.75（394） | 6.55（48） | 4.09（30） | 35.61（261） | 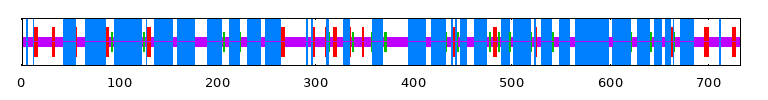 |
| *SiPUB5* | 59.58（255） | 4.67（20） | 2.57（11） | 33.18（142） | 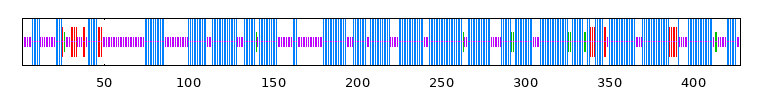 |
| *SiPUB2* | 48.31（357） | 10.42（77） | 5.55（41） | 35.72（264） | 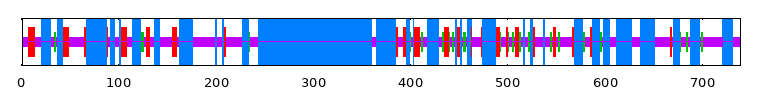 |
| *SiPUB12* | 46.90（333） | 8.59（61） | 3.80（27） | 40.70（289） | 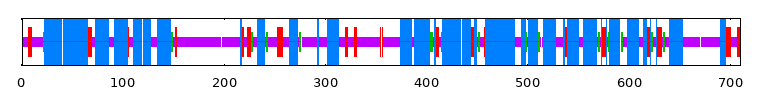 |
| *SiPUB10* | 56.67（561） | 8.48（84） | 4.14（41） | 30.71（304） | 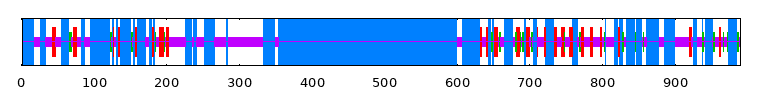 |
| *SiPUB3* | 56.67（259） | 3.72（17） | 6.35（29） | 33.26（152） | 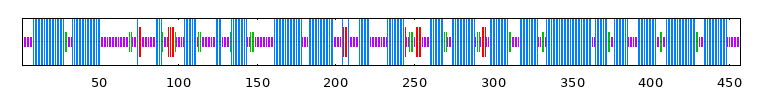 |
| *SiPUB11* | 59.67（256） | 5.13（22） | 3.73（16） | 31.47（135） | 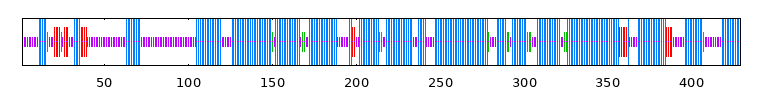 |
| *SiPUB7* | 55.15（225） | 10.05（41） | 8.33（34） | 26.47（108） | 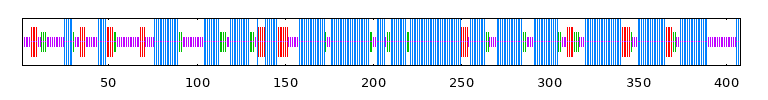 |
| *SiPUB16* | 47.91（401） | 10.87（91） | 5.26（44） | 35.96（301） | 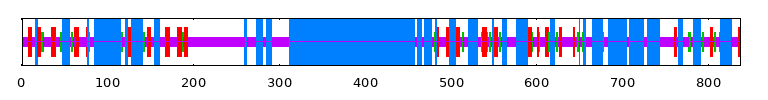 |
| *SiPUB6* | 60.23（265） | 7.05（31） | 7.73（34） | 25.00（110） | 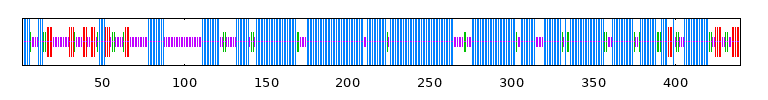 |
| *SiPUB13* | 61.18（591） | 5.18（50） | 3.93（38） | 29.71（287） | 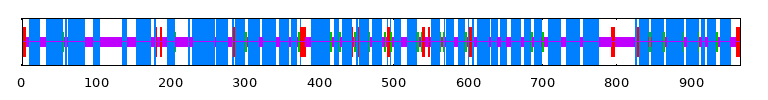 |
| *SiPUB9* | 62.94（304） | 10.97（53） | 5.59（27） | 20.50（99） | 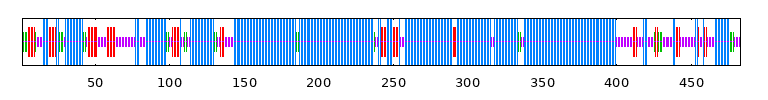 |
| *SiPUB4* | 55.18（314） | 3.69（21） | 2.64（15） | 38.49（219） | 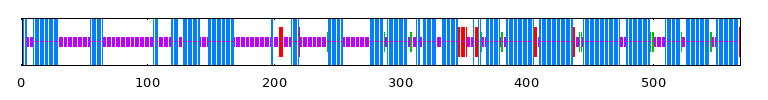 |
| *SiPUB15* | 56.39（256） | 4.85（22） | 3.52（16） | 35.24（160） | 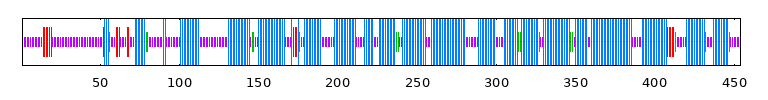 |
| *SiPUB14* | 64.99（414） | 2.83（18） | 5.34（34） | 26.84（171） | 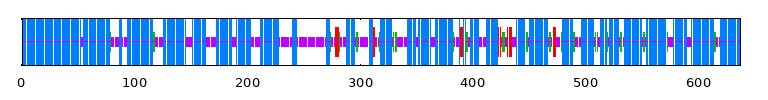 |
| *SiPUB8* | 52.05（343） | 10.02（66） | 5.77（38） | 32.17（212） | 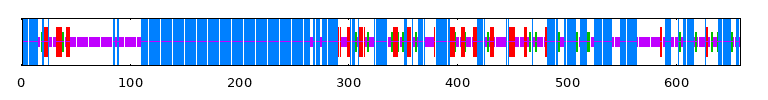 |
| *SiPUB18* | 44.35（385） | 11.75（102） | 4.61（40） | 39.29（341） | 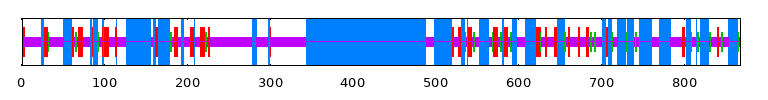 |
| *SiPUB20* | 56.17（396） | 5.96（42） | 5.25（37） | 32.62（230） | 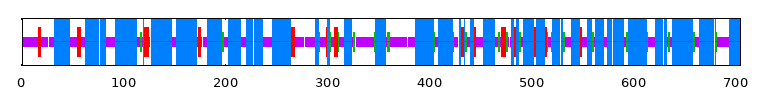 |
| *SiPUB19* | 47.36（368） | 9.27（72） | 5.28（41） | 38.10（296） | 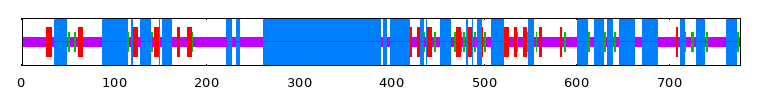 |
| *SiPUB21* | 56.76（554） | 5.74（56） | 5.43（53） | 32.07（313） | 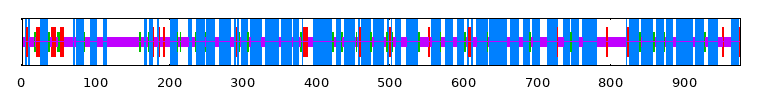 |
| *SiPUB24* | 70.86（197） | 5.40（15） | 2.16（6） | 21.58（60） | 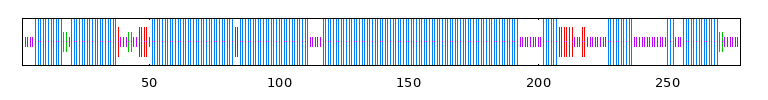 |
| *SiPUB25* | 62.56（416） | 5.41（36） | 5.56（37） | 26.47（176） | 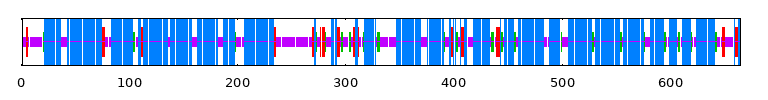 |
| *SiPUB27* | 62.72（508） | 4.20（34） | 4.69（38） | 28.40（230） | 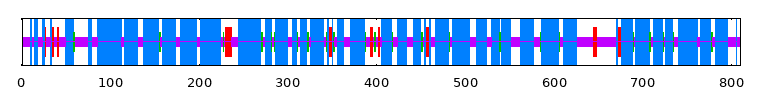 |
| *SiPUB28* | 58.47（397） | 5.15（35） | 5.60（38） | 30.78（209） | 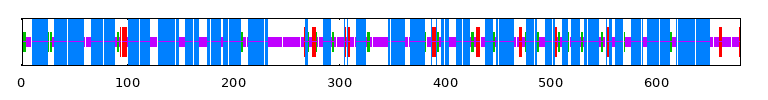 |
| *SiPUB26* | 61.06（276） | 4.65（21） | 7.30（33） | 26.99（122） | 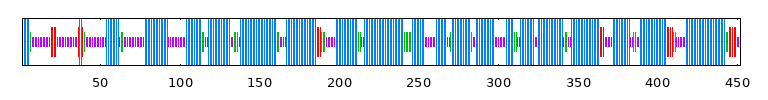 |
| *SiPUB22* | 60.58（252） | 4.09（17） | 1.44（6） | 33.89（141） | 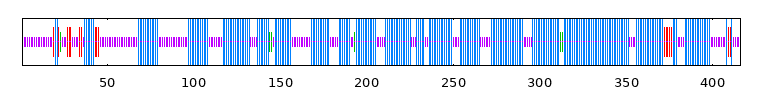 |
| *SiPUB23* | 59.81（253） | 4.26（18） | 3.07（13） | 32.86（139） | 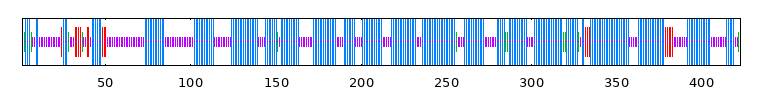 |
| *SiPUB37* | 61.21（393） | 4.05（26） | 3.89（25） | 30.84（198） | 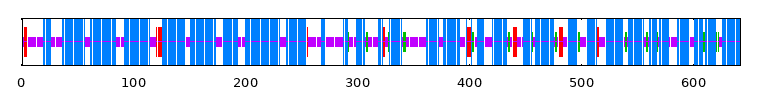 |
| *SiPUB34* | 64.06（656） | 4.88（50） | 3.81（39） | 27.25（279） | 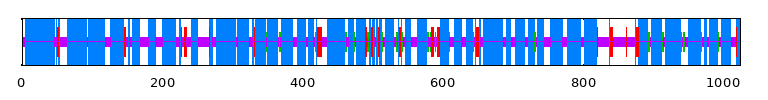 |
| *SiPUB32* | 65.30（495） | 6.99（53） | 5.01（38） | 22.69（172） | 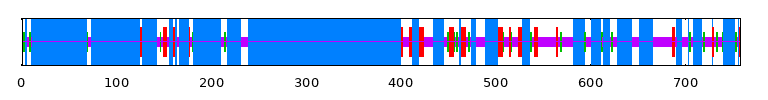 |
| *SiPUB33* | 59.56（268） | 5.33（24） | 3.11（14） | 32.00（144） | 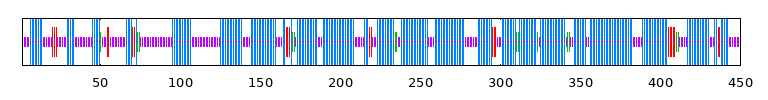 |
| *SiPUB30* | 46.88（376） | 9.23（74） | 5.24（42） | 38.65（310） | 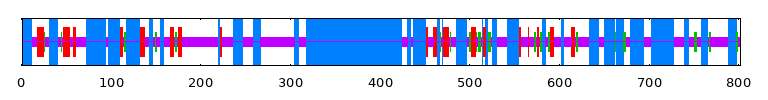 |
| *SiPUB29* | 60.25（338） | 3.39（19） | 5.53（31） | 30.84（173） | 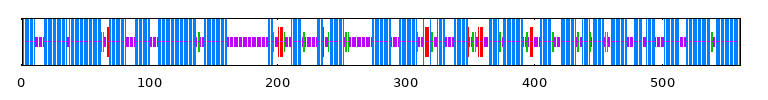 |
| *SiPUB31* | 64.32（503） | 8.18（64） | 5.63（44） | 21.87（171） | 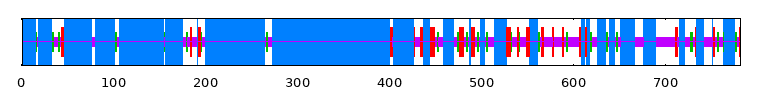 |
| *SiPUB36* | 45.22（350） | 10.47（81） | 4.52（35） | 39.79（308） | 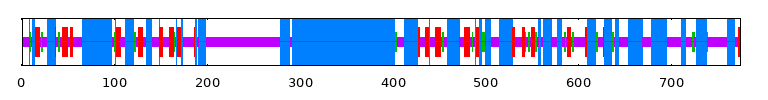 |
| *SiPUB35* | 49.22（346） | 7.68（54） | 4.13（29） | 38.98（274） | 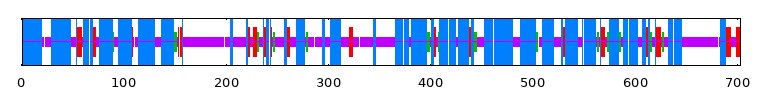 |
| *SiPUB62* | 59.77（361） | 7.12（43） | 4.30（26） | 28.81（174） | 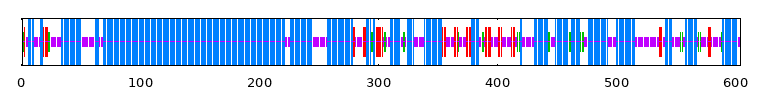 |
| *SiPUB65* | 62.29（641） | 3.98（41） | 1.36（14） | 32.36（333） | 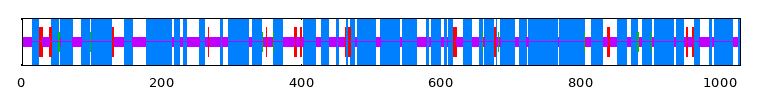 |
| *SiPUB59* | 68.63（291） | 4.72（20） | 4.01（17） | 22.64（96） | 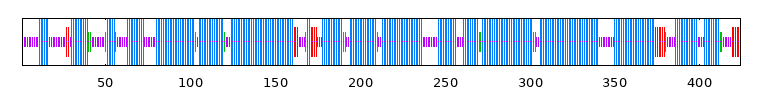 |
| *SiPUB58* | 26.97（192） | 22.47（160） | 6.04（43） | 44.52（317） | 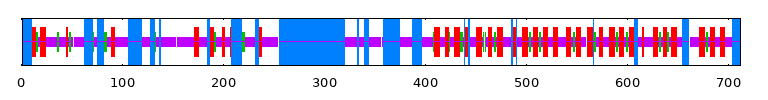 |
| *SiPUB64* | 55.73（389） | 11.03（77） | 5.16（36） | 28.08（196） | 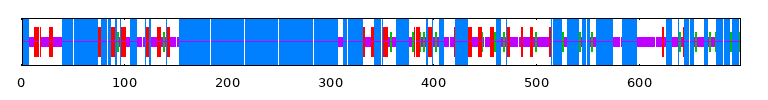 |
| *SiPUB60* | 41.98（348） | 10.49（87） | 4.95（41） | 42.58（353） | 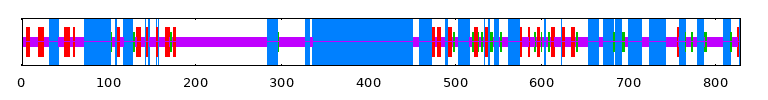 |
| *SiPUB69* | 58.61（269） | 8.28（38） | 5.23（24） | 27.89（128） | 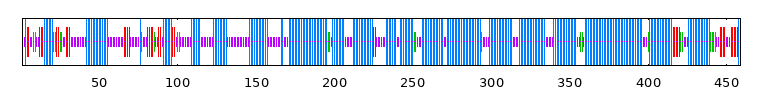 |
| *SiPUB66* | 39.33（164） | 11.75（49） | 6.71（28） | 42.21（176） | 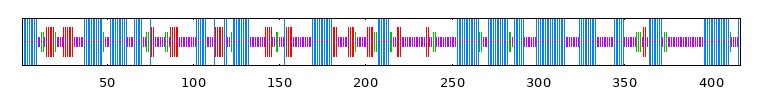 |
| *SiPUB61* | 47.23（384） | 9.72（79） | 4.80（39） | 38.25（311） | 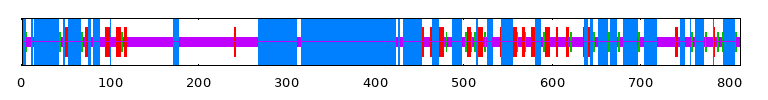 |
| *SiPUB63* | 51.24（227） | 10.61（47） | 2.71（12） | 35.44（157） | 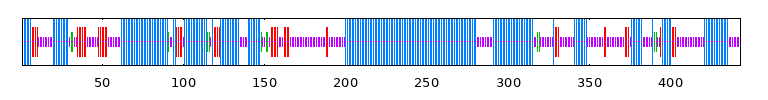 |
| *SiPUB70* | 60.45（347） | 2.79（16） | 4.01（23） | 32.75（188） | 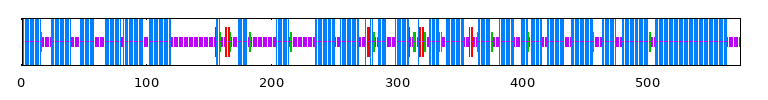 |
| *SiPUB67* | 61.42（355） | 4.50（26） | 3.81（22） | 30.28（175） | 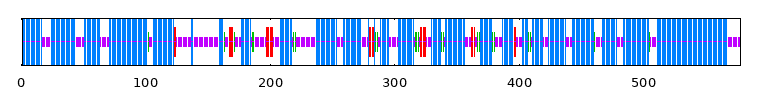 |
| *SiPUB68* | 61.94（275） | 5.41（24） | 4.05（18） | 28.60（127） | 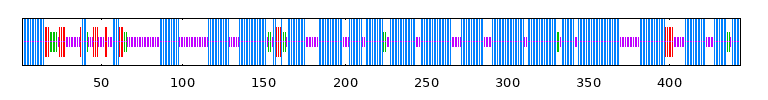 |
| *SiPUB40* | 62.75（256） | 2.70（11） | 2.21（9） | 32.35（132） | 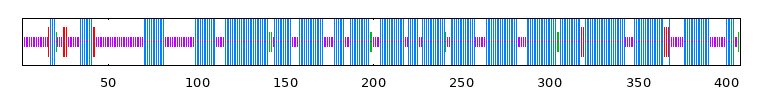 |
| *SiPUB42* | 50.56（403） | 6.27（50） | 4.52（36） | 38.64（308） | 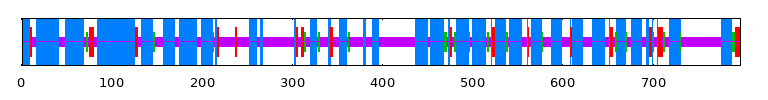 |
| *SiPUB41* | 40.88（296） | 11.60（84） | 6.49（47） | 41.02（297） | 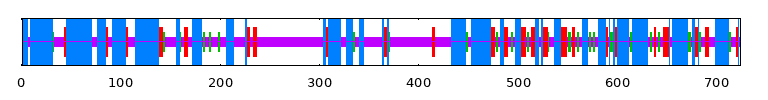 |
| *SiPUB39* | 59.34（413） | 5.46（38） | 4.74（33） | 30.46（212） | 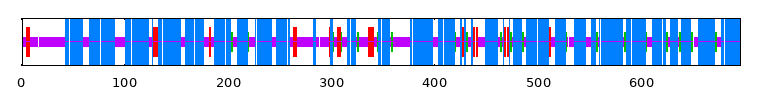 |
| *SiPUB38* | 47.48（481） | 13.92（141） | 6.12（62） | 32.48（329） | 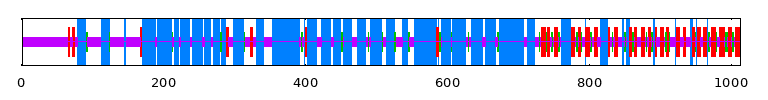 |
| *SiPUB46* | 54.46（385） | 6.36（45） | 4.67（33） | 34.51（244） | 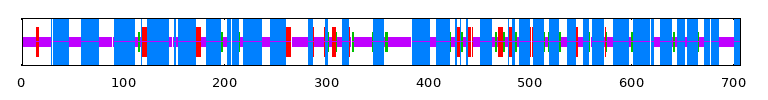 |
| *SiPUB44* | 50.56（180） | 8.71（31） | 8.71（31） | 32.02（114） | 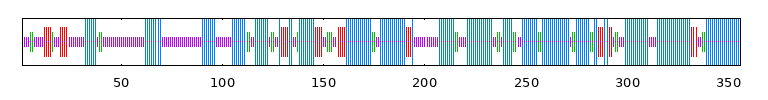 |
| *SiPUB48* | 59.09（377） | 4.23（27） | 5.02（32） | 31.66（202） | 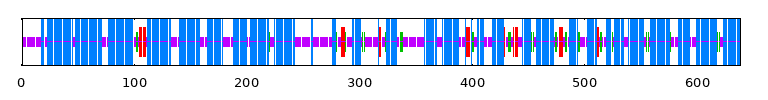 |
| *SiPUB47* | 50.66（305） | 8.31（50） | 7.64（46） | 33.39（201） | 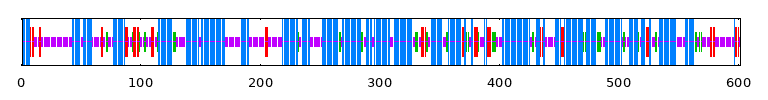 |
| *SiPUB45* | 68.36（188） | 6.91（19） | 3.27（9） | 21.45（59） | 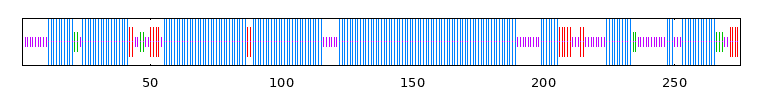 |
| *SiPUB43* | 42.22（350） | 7.96（66） | 4.83（40） | 44.99（373） | 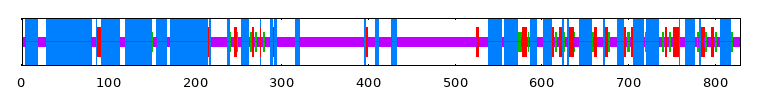 |
| *SiPUB51* | 55.05（218） | 5.05（20） | 4.80（19） | 35.10（139） | 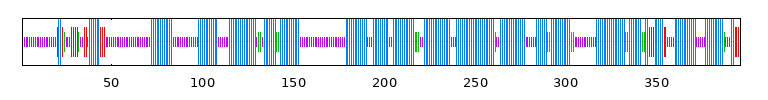 |
| *SiPUB54* | 52.83（438） | 5.79（48） | 4.22（35） | 37.15（308） | 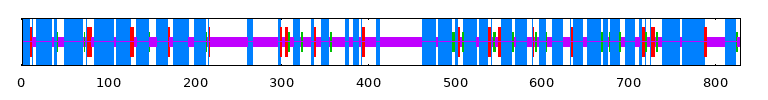 |
| *SiPUB49* | 52.90（246） | 5.38（25） | 6.88（32） | 34.84（162） | 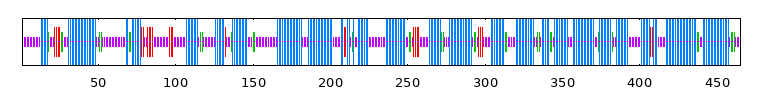 |
| *SiPUB57* | 61.31（263） | 4.43（19） | 3.03（13） | 31.24（134） | 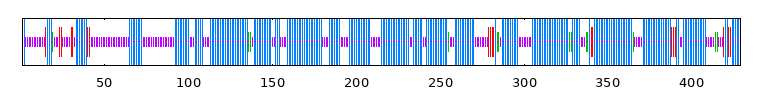 |
| *SiPUB50* | 46.13（316） | 8.03（55） | 6.42（44） | 39.42（270） | 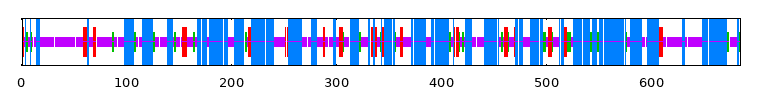 |
| *SiPUB53* | 42.67（323） | 10.30（78） | 5.28（40） | 41.74（316） | 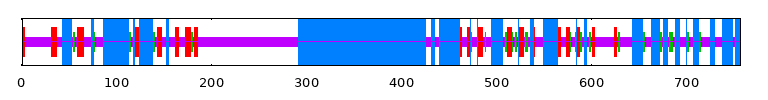 |
| *SiPUB55* | 61.29（521） | 4.59（39） | 4.24（36） | 29.88（254） | 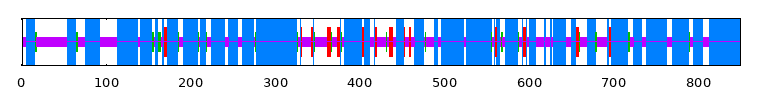 |
| *SiPUB56* | 61.74（255） | 3.15（13） | 3.39（14） | 31.72（131） | 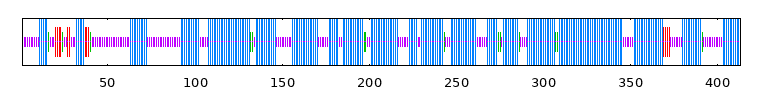 |
| *SiPUB52* | 58.37（258） | 6.11（27） | 6.33（28） | 29.19（129） | 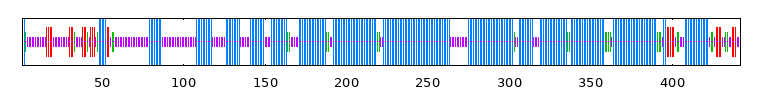 |

Alpha-helix(Blue); Beta-sheet(Green); Random coil(Yellow); Extended strand(Red).
